# Supplementary material for: Base editing-mediated one-step inactivation of the Dnmt gene family reveals critical roles of DNA methylation during mouse gastrulation
Source: Nat Commun. 2023 May 22;14:2922. doi: 10.1038/s41467-023-38528-z (PMC10203112; doi:10.1038/s41467-023-38528-z)
Supplement: Supplementary file 1 — Supplementary Information [file 41467_2023_38528_MOESM1_ESM.pdf]

*Supplemental material*

# Base editing-mediated one-step inactivation of the *Dnmt* gene family reveals critical roles of DNA methylation during mouse gastrulation

Qing Li[1,#], Jiansen Lu[2,#], Xidi Yin[1,#], Yunjian Chang[3,#], Chao Wang[3,#], Meng Yan[4,#], Li Feng[5,6,7], Yanbo Cheng[8], Yun Gao[2], Beiying Xu[3], Yao Zhang[3], Yingyi Wang[8], Guizhong Cui[1], Luang Xu[3], Yidi Sun[5], Rong Zeng[6], Yixue Li[7], Naihe Jing[1], Guo-Liang Xu[3,*], Ligang Wu[3,*], Fuchou Tang[2,*] & Jinsong Li[1,4,8,*]

[1]State Key Laboratory of Cell Biology, Shanghai Key Laboratory of Molecular Andrology, Shanghai Institute of Biochemistry and Cell Biology, Center for Excellence in Molecular Cell Science, Chinese Academy of Sciences, University of Chinese Academy of Sciences, Shanghai, China.
[2]School of Life Sciences, Biomedical Pioneering Innovation Center, Beijing Advanced Innovation Center for Genomics, Peking University, Beijing, China.
[3]State Key Laboratory of Molecular Biology, Shanghai Key Laboratory of Molecular Andrology, Shanghai Institute of Biochemistry and Cell Biology, Center for Excellence in Molecular Cell Science, Chinese Academy of Sciences, Shanghai, China.
[4]School of Life Science, Hangzhou Institute for Advanced Study, University of Chinese Academy of Sciences, Hangzhou, China.
[5]Institute of Neuroscience, CAS Center for Excellence in Brain Science and Intelligence Technology, Chinese Academy of Sciences, Shanghai, China.
[6]CAS Key Laboratory of Systems Biology, Center for Excellence in Molecular Cell Science, Institute of Biochemistry and Cell Biology, Chinese Academy of Sciences, Shanghai, China.
[7]Bio-Med Big Data Center, Key Laboratory of Computational Biology, CAS-MPG Partner Institute for Computational Biology, Shanghai Institute of Nutrition and Health, Chinese Academy of Sciences, Shanghai, China.
[8]School of Life Science and Technology, Shanghai Tech University, Shanghai, China.
[#]These authors contributed equally: Qing Li, Jiansen Lu, Xidi Yin, Yunjian Chang, Chao Wang, Meng Yan.
*e-mail: glxu@sibcb.ac.cn; lgwu@sibcb.ac.cn; tangfuchou@pku.edu.cn; jsli@sibcb.ac.cn.

**Supplementary Materials**

Supplementary Figs. 1-19

Supplementary References

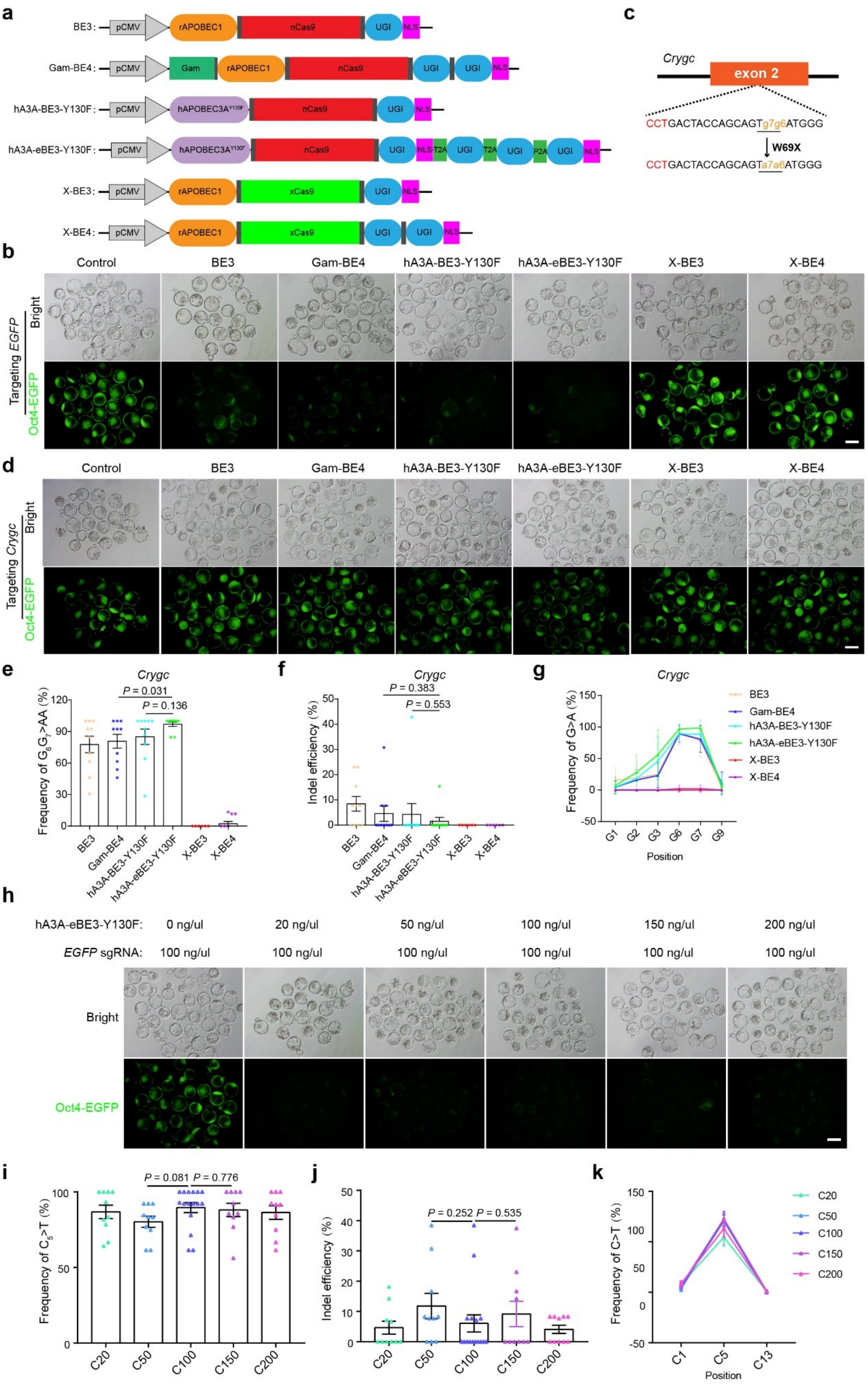

a
BE3 : pCMV rAPOBEC1 nCas9 UGI NLS
Gam-BE4 : pCMV Gam rAPOBEC1 nCas9 UGI UGI NLS
hA3A-BE3-Y130F : pCMV hAPOBEC3A nCas9 UGI NLS
hA3A-eBE3-Y130F : pCMV hAPOBEC3A nCas9 UGI NLS T2A UGI T2A UGI P2A UGI NLS
X-BE3 : pCMV rAPOBEC1 xCas9 UGI NLS
X-BE4 : pCMV rAPOBEC1 xCas9 UGI UGI NLS
c
Crygc
exon 2
CCTGACTACCAGCAGTg7g6ATGGG
W69X
CCTGACTACCAGCAGTa7a6ATGGG
b
Control
BE3
Gam-BE4
hA3A-BE3-Y130F
hA3A-eBE3-Y130F
X-BE3
X-BE4
Targeting EGFP
Bright
Oct4-EGFP
d
Targeting Crygc
e
Crygc
Frequency of G6G7>AA (%)
P = 0.031
P = 0.136
f
Indel efficiency (%)
P = 0.383
P = 0.553
g
Frequency of G>A (%)
Position
G1 G2 G3 G6 G7 G9
h
hA3A-eBE3-Y130F: 0 ng/ul 20 ng/ul 50 ng/ul 100 ng/ul 150 ng/ul 200 ng/ul
EGFP sgRNA: 100 ng/ul 100 ng/ul 100 ng/ul 100 ng/ul 100 ng/ul 100 ng/ul
i
Frequency of C5>T (%)
P = 0.081 P = 0.776
C20 C50 C100 C150 C200
j
Indel efficiency (%)
P = 0.252 P = 0.535
k
Frequency of C>T (%)
Position
C1 C5 C13

**Supplementary Fig. 1 | hA3A-eBE3-Y130F is the most efficient base editor for inducing stop codon in zygotes.**

**a,** Schematic illustration of six reported base editors, including BE3, Gam-BE4, hA3A-BE3-Y130F, hA3A-eBE3-Y130F, X-BE3, and X-BE4. **b,** Representative images of blastocysts obtained by injection of base-editor mRNA and sgRNA targeting *EGFP* into zygotes carrying *Oct4-EGFP* transgene. Three independent experiments were analyzed for each group. Scale bar, 200 µm. **c,** The target sequence in *Crygc*. The PAM sequence and the intended mutant bases are shown in red and yellow, respectively. **d,** Representative images of blastocysts obtained by injection of base-editor mRNA and sgRNA targeting *Crygc* into zygotes carrying *Oct4-EGFP* transgene. Three independent experiments were analyzed for each group. Scale bar, 200 µm. **e, f, g,** Efficiency analysis of on-target G-to-A base conversion (**e**), indels (**f**), and adjacent site mutation (**g**) induced by different base editors in *Crygc*. Data are mean ± s.e.m for the indicated numbers of blastocysts. Detailed data are shown in Supplementary Data 1. *P*-values were determined by Student's unpaired two-sided *t*-test. **h,** Representative images of blastocysts obtained by injection of hA3A-eBE3-Y130F mRNA with different concentrations (including 0 ng/µl, 20 ng/µl, 50 ng/µl, 100 ng/µl, 150 ng/µl, and 200 ng/µl) and sgRNA targeting *EGFP* (100 ng/µl) into zygotes carrying *Oct4-EGFP* transgene. Three independent experiments were analyzed for each group. Scale bar, 100 µm. **i, j, k,** Statistical analysis of on-target C-to-T base conversions (**i**), indels (**j**), and adjacent site mutation (**k**) in *EGFP* site induced by hA3A-eBE3-Y130F mRNA with different concentrations (C20, *n* = 10; C50, *n* = 10; C100, *n* = 16; C150, *n* = 10; C200, *n* = 10;) and *EGPF*-sgRNA in blastocysts. Detailed data are shown in Supplementary Data 1. *n*, the number of collected blastocysts. Data are mean ± s.e.m for the indicated numbers of blastocysts. *P*-values were determined by Student's unpaired two-sided *t*-test. All source data are provided as a Source Data file.

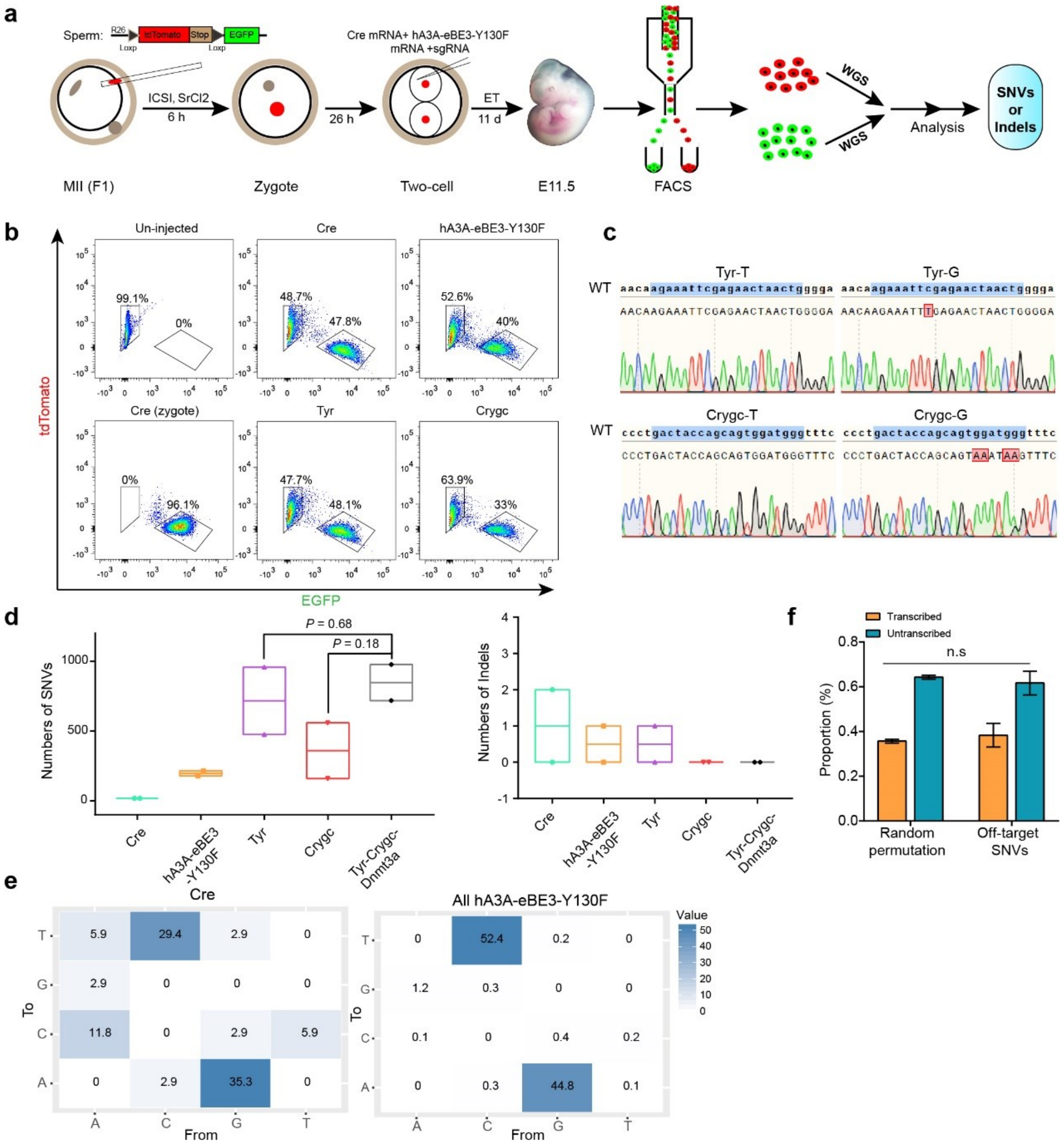
a
Sperm:
R26
Loxp
tdTomato
Stop
EGFP
Loxp
ICSI, SrCl2
6 h
26 h
Cre mRNA+ hA3A-eBE3-Y130F mRNA +sgRNA
ET
11 d
WGS
WGS
Analysis
SNVs or Indels
MII (F1)
Zygote
Two-cell
E11.5
FACS
b
Un-injected
Cre
hA3A-eBE3-Y130F
99.1%
0%
48.7%
47.8%
52.6%
40%
Cre (zygote)
Tyr
Crygc
0%
96.1%
47.7%
48.1%
63.9%
33%
tdTomato
EGFP
c
Tyr-T
Tyr-G
WT
aacaagaaattcgagaactaactgggga
AACAAGAAATTCGAGAACTAACTGGGGA
Crygc-T
Crygc-G
WT
ccctgactaccagcagtggatgggtttc
CCCTGACTACCAGCAGTGGATGGGTTTC
CCCTGACTACCAGCAGTAAATAAGTTTC
d
P = 0.68
P = 0.18
Numbers of SNVs
Numbers of Indels
Cre
hA3A-eBE3 -Y130F
Tyr
Crygc
Tyr-Crygc-Dnmt3a
f
Transcribed
Untranscribed
n.s
Proportion (%)
Random permutation
Off-target SNVs
e
Cre
All hA3A-eBE3-Y130F
Value
To
From
T
G
C
A
5.9
29.4
2.9
0
2.9
0
0
0
11.8
0
2.9
5.9
0
2.9
35.3
0
0
52.4
0.2
0
1.2
0.3
0
0
0.1
0
0.4
0.2
0
0.3
44.8
0.1

**Supplementary Fig. 2 | GOIT analysis reveals minor and random off-target mutations induced by hA3A-eBE3-Y130F.**

**a,** Experimental procedure of GOIT (genome-wide off-target analysis by two-cell embryo injection) to detect off-target of hA3A-eBE3-Y130F. **b,** Representative images of FACS gating strategy for collection of EGFP positive cells and tdTomato positive cells from E11.5 embryos derived by injection of RNAs into one blastomere of the two-cell embryos. Four RNA injection groups (two repeats for each one) include Cre mRNA only (Cre), mRNA of Cre and hA3A-eBE3-Y130F, mRNA of Cre and hA3A-eBE3-Y130F and *Tyr*-sgRNA (Tyr), and mRNA of Cre and hA3A-eBE3-Y130F and *Crygc*-sgRNA (Crygc). EGFP positive cells are derived from injected blastomere and tdTomato positive cells are derived from un-injected blastomere. The enriched cells were used for whole genome sequencing (WGS) analysis. **c,** Sanger sequencing of DNA from tdTomato positive cells (T) and EGFP positive cells (G) in Tyr and Crygc groups, indicating that tdTomato positive cells are wild-type and EGFP positive cells harbor mutant *Try* or *Crygc*. **d,** Total number of off-target SNVs and indels detected by whole genome sequencing (WGS) in E11.5 embryos from five groups. Try-Crygc-Dnmt3a group means injecting a mixture of Cre and hA3A-eBE3-Y130F mRNA with *Try*, *Crygc, and Dnmt3a* sgRNAs. *P*-values were determined by Student's unpaired two-sided *t*-test. Floating bars show minima to maxima from two biological replicates. Lists of off-target SNVs and indels are presented in Supplementary Data 2. **e,** Mutation type distributions of SNVs shown in (**d**). The number in each cell indicates the proportion of a certain type of mutation among all mutations, indicating that more than 97% of SNVs are C>T/G>A base conversions induced by hA3A-eBE3-Y130F. **f,** Genomic distribution of off-target SNVs induced by hA3A-eBE3-Y130F ($n$ = 2580) or random permutation ($n$ = 3.0E7) according to transcription and non-transcription regions, showing no significant difference between hA3A-eBE3-Y130F-induced SNVs and random permutation. $n$ means the number of SNV sites. Data are mean ± SD. *P*-value =1 was calculated by the two-sided Fisher's exact test. All source data are provided as a Source Data file.

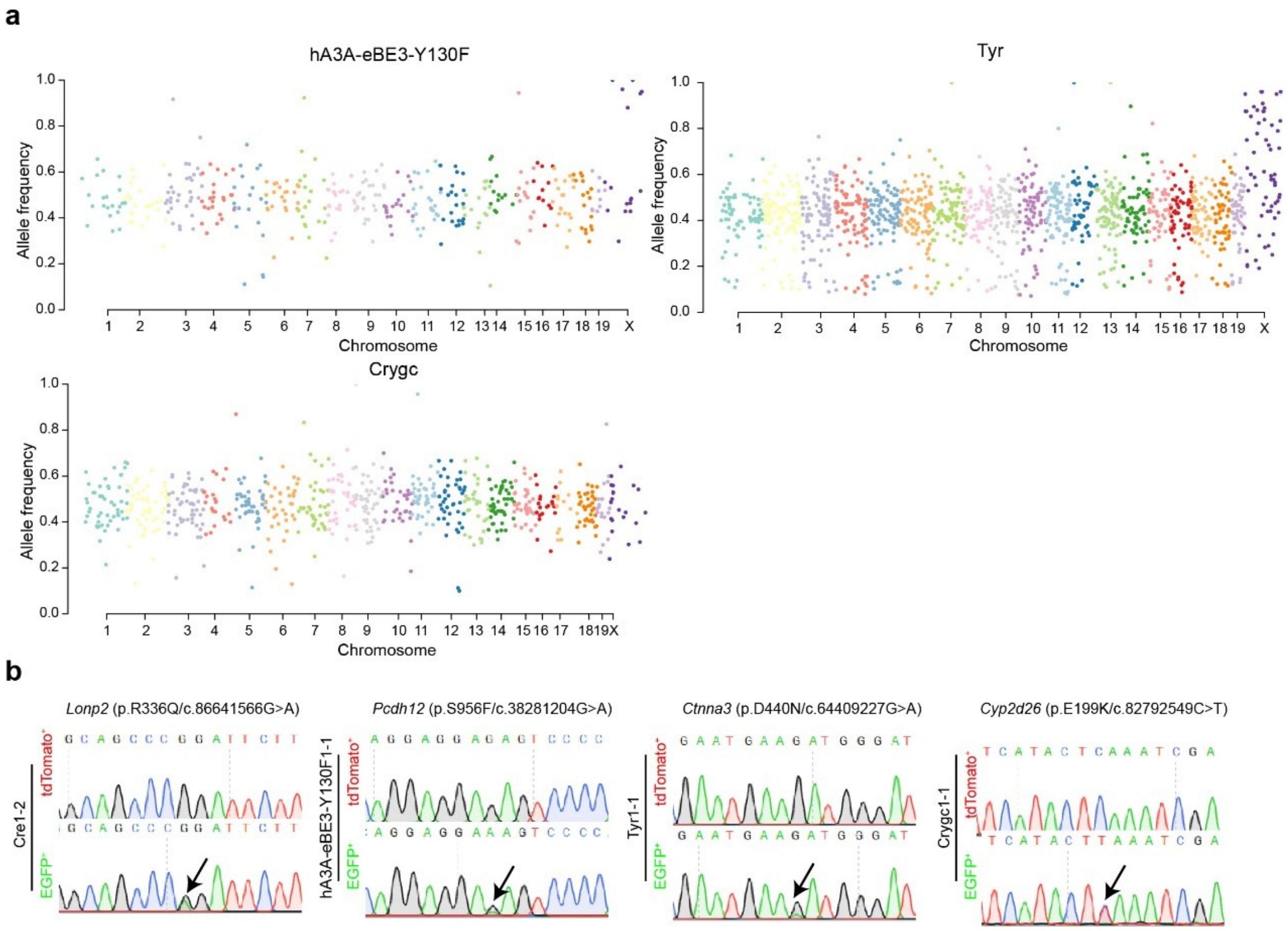


**Supplementary Fig. 3 | The distribution of off-target mutations in the genome.**

**a,** The Manhattan plot shows the distribution of all off-target mutations of different groups (hA3A-eBE3-Y130F, Tyr, and Crygc) in the genome. The abscissa is the location of the mutations on the chromosome, and the ordinate is the frequency of each mutation. Each point presents a mutation (SNV or indel), and the different colors indicate different chromosomes. **b,** Representative Sanger sequence chromatograms show the verification results of non-synonymous off-target mutations in each group. Summary of the verification was shown in Supplementary Data 2. Black arrows indicate the mutant sites. All check primers are listed in Supplementary Data 9. All source data are provided as a Source Data file.

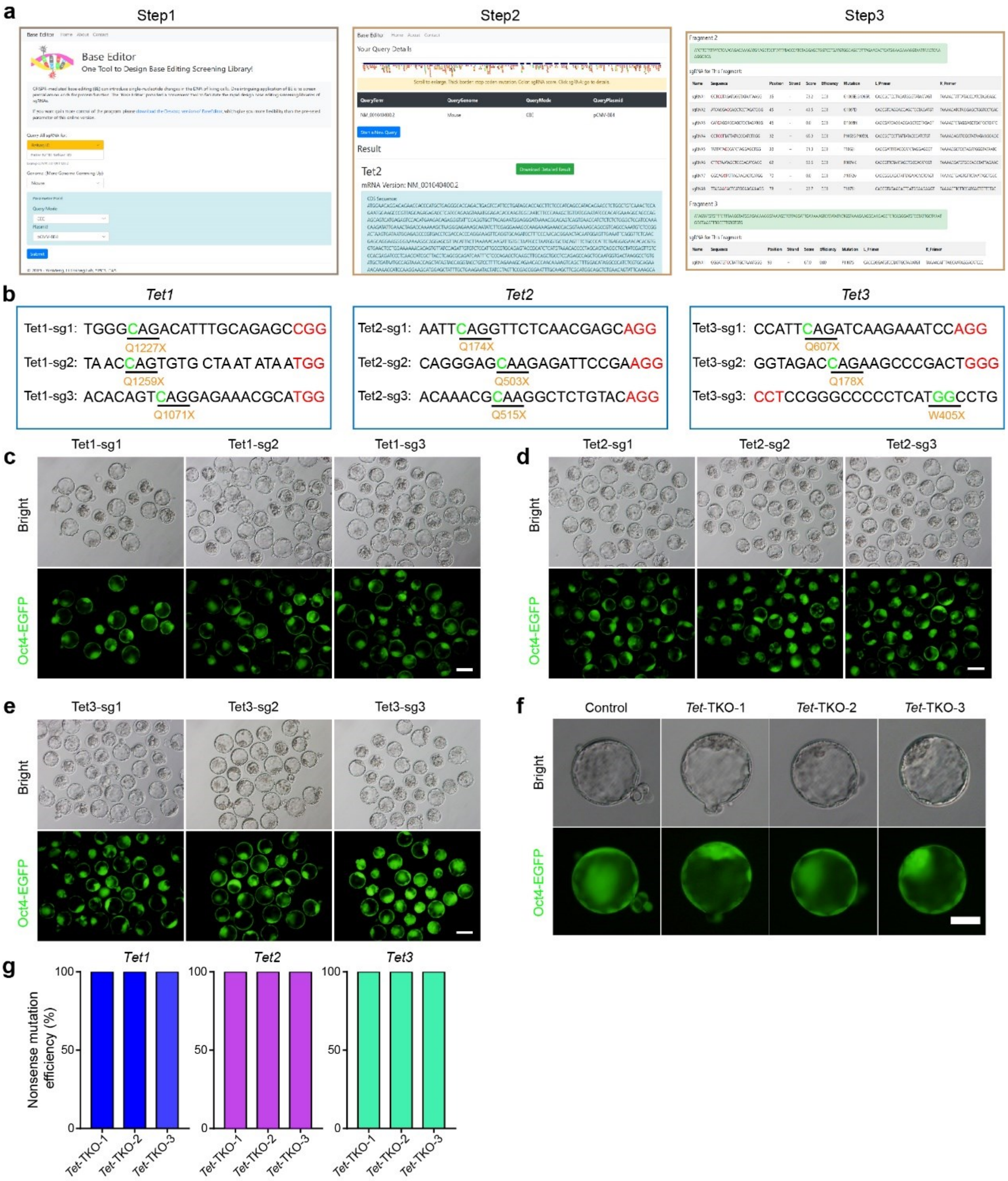
a
Step1
Step2
Step3
b
Tet1
Tet2
Tet3
Tet1-sg1: TGGGCAGACATTTGCAGAGCCGG
Q1227X
Tet1-sg2: TA ACCAGTGTG CTA AT ATA ATGG
Q1259X
Tet1-sg3: ACACAGTCAGGAGAAACGCATGG
Q1071X
Tet2-sg1: AATTCAGGTTCTCAACGAGCAGG
Q174X
Tet2-sg2: CAGGGAGCAAGAGATTCCGAAGG
Q503X
Tet2-sg3: ACAAACGCAAGGCTCTGTACAGG
Q515X
Tet3-sg1: CCATTCAGATCAAGAAATCCAGG
Q607X
Tet3-sg2: GGTAGACCAGAAGCCCGACTGGG
Q178X
Tet3-sg3: CCTCCGGGCCCCCTCATGGCCTG
W405X
c
Tet1-sg1
Tet1-sg2
Tet1-sg3
Bright
Oct4-EGFP
d
Tet2-sg1
Tet2-sg2
Tet2-sg3
e
Tet3-sg1
Tet3-sg2
Tet3-sg3
f
Control
Tet-TKO-1
Tet-TKO-2
Tet-TKO-3
g
Nonsense mutation efficiency (%)
100
50
0
Tet-TKO-1
Tet-TKO-2
Tet-TKO-3

**Supplementary Fig. 4 | One-step generation of *Tet*-TKO embryos by IMGZ.**
**a,** Schematic of the procedure to design sgRNAs to target *Tet2* using Base-Editor. Base-Editor website has been established to design sgRNAs of cytosine base editors to induce stop codons in a specific gene. Coded by C# ASP.NET. Gene name or Refseq ID is required for sgRNA designing. **b,** Sequence of sgRNAs designed by Base-Editor for targeting *Tet1*, *Tet2*, and *Tet3*, respectively. The PAM sequence and the intended mutant base are shown in red and green, respectively. **c, d, e,** Representative images of blastocysts obtained by injection of hA3A-eBE3-Y130F mRNA and different sgRNAs targeting *Tet1* (**c**), *Tet2* (**d**), and *Tet3* (**e**), respectively, into zygotes carrying *Oct4-EGFP* transgene. Scale bar, 100 μm. Three independent experiments were analyzed for each group. **f,** Representative images of *Tet1/2/3*-TKO blastocysts. Green fluorescence indicates the expression of *Oct4-EGFP*. Scale bar, 50 μm. Three independent embryos were analyzed for each group. **g,** The editing efficiencies of mutant sites in three *Tet1/2/3*-TKO blastocysts detected by blunting cloning sequencing. All source data are provided as a Source Data file.

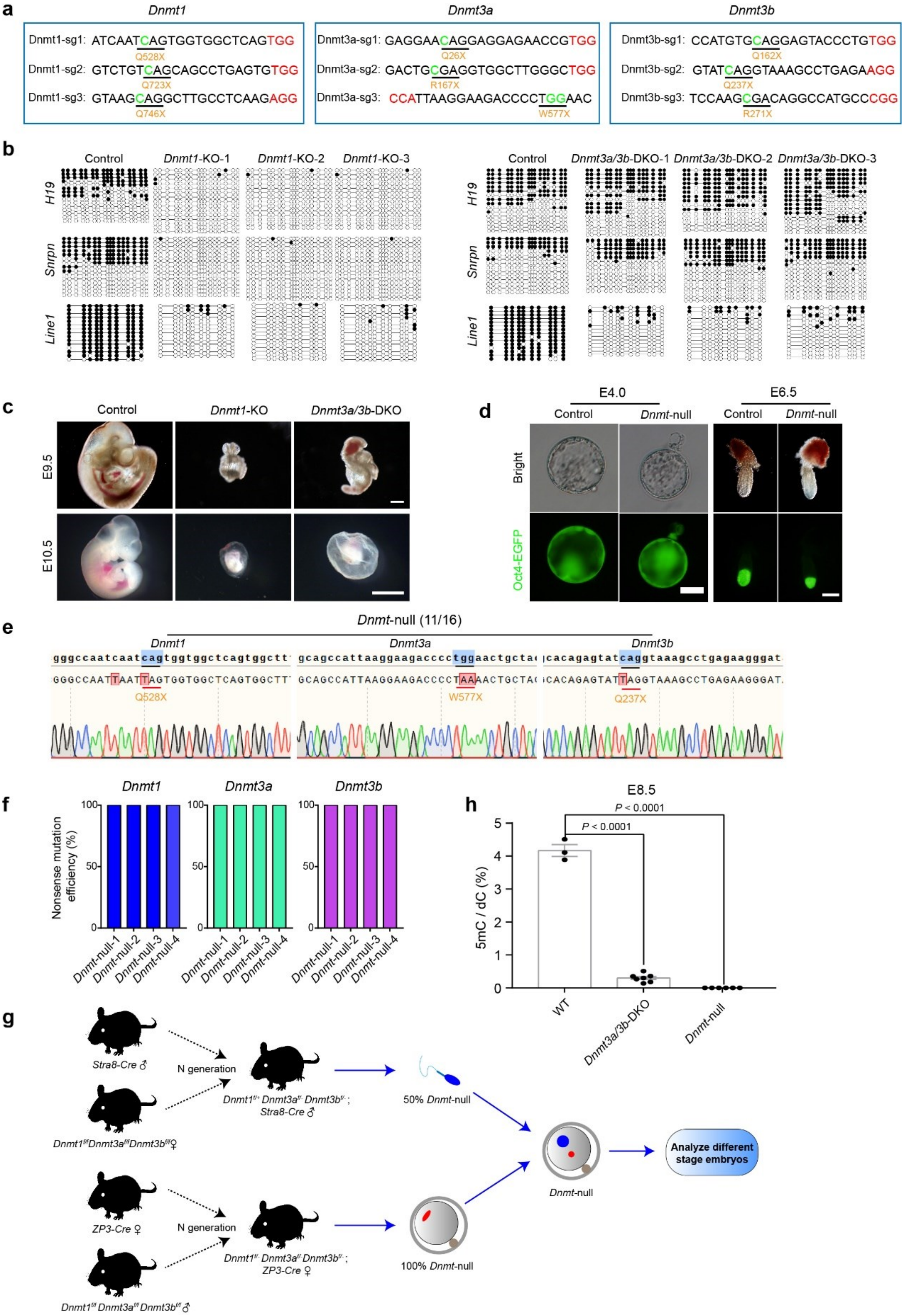

a
Dnmt1
Dnmt1-sg1: ATCAATCAGTGGTGGCTCAGTGG
Q528X
Dnmt1-sg2: GTCTGTCAGCAGCCTGAGTGTGG
Q723X
Dnmt1-sg3: GTAAGCAGGCTTGCCTCAAGAGG
Q746X
Dnmt3a
Dnmt3a-sg1: GAGGAACAGGAGGAGAACCGTGG
Q26X
Dnmt3a-sg2: GACTGCGAGGTGGCTTGGGCTGG
R167X
Dnmt3a-sg3: CCATTAAGGAAGACCCCTGGAAC
W577X
Dnmt3b
Dnmt3b-sg1: CCATGTGCAGGAGTACCCTGTGG
Q162X
Dnmt3b-sg2: GTATCAGGTAAAGCCTGAGAAGG
Q237X
Dnmt3b-sg3: TCCAAGCGACAGGCCATGCCCGG
R271X
b
Control
Dnmt1-KO-1
Dnmt1-KO-2
Dnmt1-KO-3
Dnmt3a/3b-DKO-1
Dnmt3a/3b-DKO-2
Dnmt3a/3b-DKO-3
H19
Snrpn
Line1
c
Control
Dnmt1-KO
Dnmt3a/3b-DKO
E9.5
E10.5
d
E4.0
E6.5
Control
Dnmt-null
Bright
Oct4-EGFP
e
Dnmt-null (11/16)
Dnmt1
Dnmt3a
Dnmt3b
gggccaatcaatcagtggtggctcagtggctt
gcagccattaaggaagacccctggaactgcta
cacagagtatcaggtaaagcctgagaagggat
GGGCCAATTAATTAGTGGTGGCTCAGTGGCTT
GCAGCCATTAAGGAAGACCCCTAAAACTGCTA
CACAGAGTATTAGGTAAAGCCTGAGAAGGGAT
Q528X
W577X
Q237X
f
Dnmt1
Dnmt3a
Dnmt3b
Nonsense mutation efficiency (%)
100
50
0
Dnmt-null-1
Dnmt-null-2
Dnmt-null-3
Dnmt-null-4
g
Stra8-Cre ♂
Dnmt1f/f Dnmt3af/f Dnmt3bf/f ♀
N generation
Dnmt1f/+ Dnmt3af/ Dnmt3bf/ ; Stra8-Cre ♂
50% Dnmt-null
ZP3-Cre ♀
Dnmt1f/f Dnmt3af/f Dnmt3bf/f ♂
Dnmt1f/ Dnmt3af/ Dnmt3bf/ ; ZP3-Cre ♀
100% Dnmt-null
Dnmt-null
Analyze different stage embryos
h
E8.5
P < 0.0001
P < 0.0001
5mC / dC (%)
5
4
3
2
1
0
WT
Dnmt3a/3b-DKO
Dnmt-null

**Supplementary Fig. 5 | Generation of *Dnmt1*-KO, *Dnmt3a/3b*-DKO, and *Dnmt*-null embryos by the IMGZ system and mating.**

**a,** Sequence of sgRNAs designed by Base-Editor for targeting *Dnmt1*, *Dnmt3a*, and *Dnmt3b*, respectively. The PAM sequence and the intended mutant base are shown in red and green, respectively. **b,** Methylation analysis of *H19*-DMR, *Snrpn*-DMR, and *Line1* promoter in *Dnmt1*-KO and *Dnmt3a/3b*-DKO embryos. DNA was extracted from E9.5 embryos. Open circles represent unmethylated CpG sites, whereas filled circles represent methylated CpG sites. **c,** Representative images of *Dnmt1*-KO and *Dnmt3a/3b*-DKO embryos at E9.5 and E10.5 generated by the IMGZ system. Three independent embryos were analyzed for each group. Scale bars, 500 µm in the up panel and 2 mm in the down panel. **d,** Representative images of *Dnmt*-null blastocysts and E6.5 embryos. Green fluorescence indicates the expression of *Oct4-EGFP*. Three independent embryos were analyzed for each group. Scale bar, 50 µm in the left panel and 100 µm in the right panel. **e,** Representative Sanger sequence chromatograms of one *Dnmt*-null blastocyst. 11 of 16 checked blastocysts show homozygous mutant chromatograms. **f,** The editing efficiencies of mutant sites in four *Dnmt*-null blastocysts detected by blunting cloning sequencing. **g,** Strategy of generating *Dnmt*-null embryos using germline-specific conditional knockout parents *(Stra8-Cre* and *ZP3-Cre*). **h,** Frequencies of 5mC modified nucleotides in the genomic DNA of wild-type ($n$ = 3), *Dnmt3a/3b*-DKO ($n$ = 7) and *Dnmt*-null ($n$ = 6) embryos at E8.5 determined by quantitative mass spectrometry. Data are mean ± s.e.m for the indicated biological replicates. $P$-values were determined by Student's unpaired two-sided $t$-test. All source data are provided as a Source Data file.

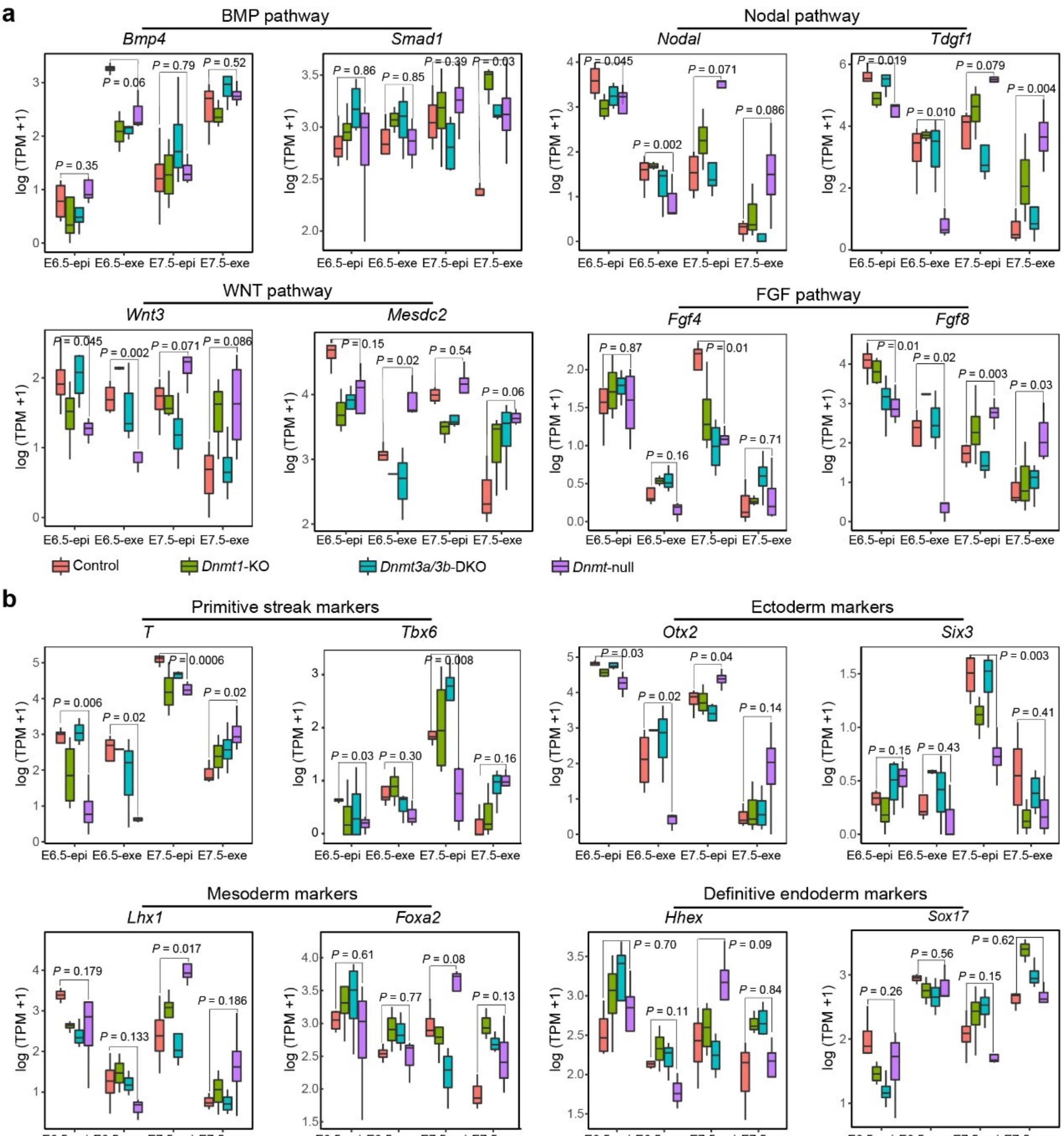
a
BMP pathway
Bmp4
Smad1
Nodal pathway
Nodal
Tdgf1
WNT pathway
Wnt3
Mesdc2
FGF pathway
Fgf4
Fgf8
log (TPM +1)
E6.5-epi
E6.5-exe
E7.5-epi
E7.5-exe
P = 0.35
P = 0.06
P = 0.79
P = 0.52
P = 0.86
P = 0.85
P = 0.39
P = 0.03
P = 0.045
P = 0.002
P = 0.071
P = 0.086
P = 0.019
P = 0.010
P = 0.079
P = 0.004
P = 0.045
P = 0.002
P = 0.071
P = 0.086
P = 0.15
P = 0.02
P = 0.54
P = 0.06
P = 0.87
P = 0.16
P = 0.01
P = 0.71
P = 0.01
P = 0.02
P = 0.003
P = 0.03
Control
Dnmt1-KO
Dnmt3a/3b-DKO
Dnmt-null
b
Primitive streak markers
T
Tbx6
Ectoderm markers
Otx2
Six3
P = 0.006
P = 0.02
P = 0.0006
P = 0.02
P = 0.03
P = 0.30
P = 0.008
P = 0.16
P = 0.03
P = 0.02
P = 0.04
P = 0.14
P = 0.15
P = 0.43
P = 0.003
P = 0.41
Mesoderm markers
Lhx1
Foxa2
Definitive endoderm markers
Hhex
Sox17
P = 0.179
P = 0.133
P = 0.017
P = 0.186
P = 0.61
P = 0.77
P = 0.08
P = 0.13
P = 0.70
P = 0.11
P = 0.09
P = 0.84
P = 0.26
P = 0.56
P = 0.15
P = 0.62
Control
Dnmt1-KO
Dnmt3a/3b-DKO
Dnmt-null

**Supplementary Fig. 6 | RNA-seq reveals impaired gastrulation-related pathways in *Dnmt*-null embryos.**

**a,** Box plots show the expression of critical genes involved in some canonical signaling pathways related to gastrulation and primitive streak formation in mice, including BMP, Nodal, WNT, and FGF pathways, in Exe and Epi at E6.5 and E7.5. The central lines are the median of data. The lower and upper hinges correspond to the 25th and 75th percentiles. The end of the lower and upper whiskers are 1.5 * IQR (inter-quartile range). Data beyond the end of the whiskers are plotted as outliers. *P*-values were determined by unpaired two-sided *t*-test. **b,** Box plots show the expression of some lineage-specific markers, including primitive streak, ectoderm, mesoderm, and definitive endoderm, in Exe and Epi at E6.5 and E7.5. The central lines are the median of data. The lower and upper hinges correspond to the 25th and 75th percentiles. The end of the lower and upper whiskers are 1.5 * IQR (inter-quartile range). Data beyond the end of the whiskers are plotted as outliers. *P*-values were determined by unpaired two-sided *t*-test. All source data are provided as a Source Data file.

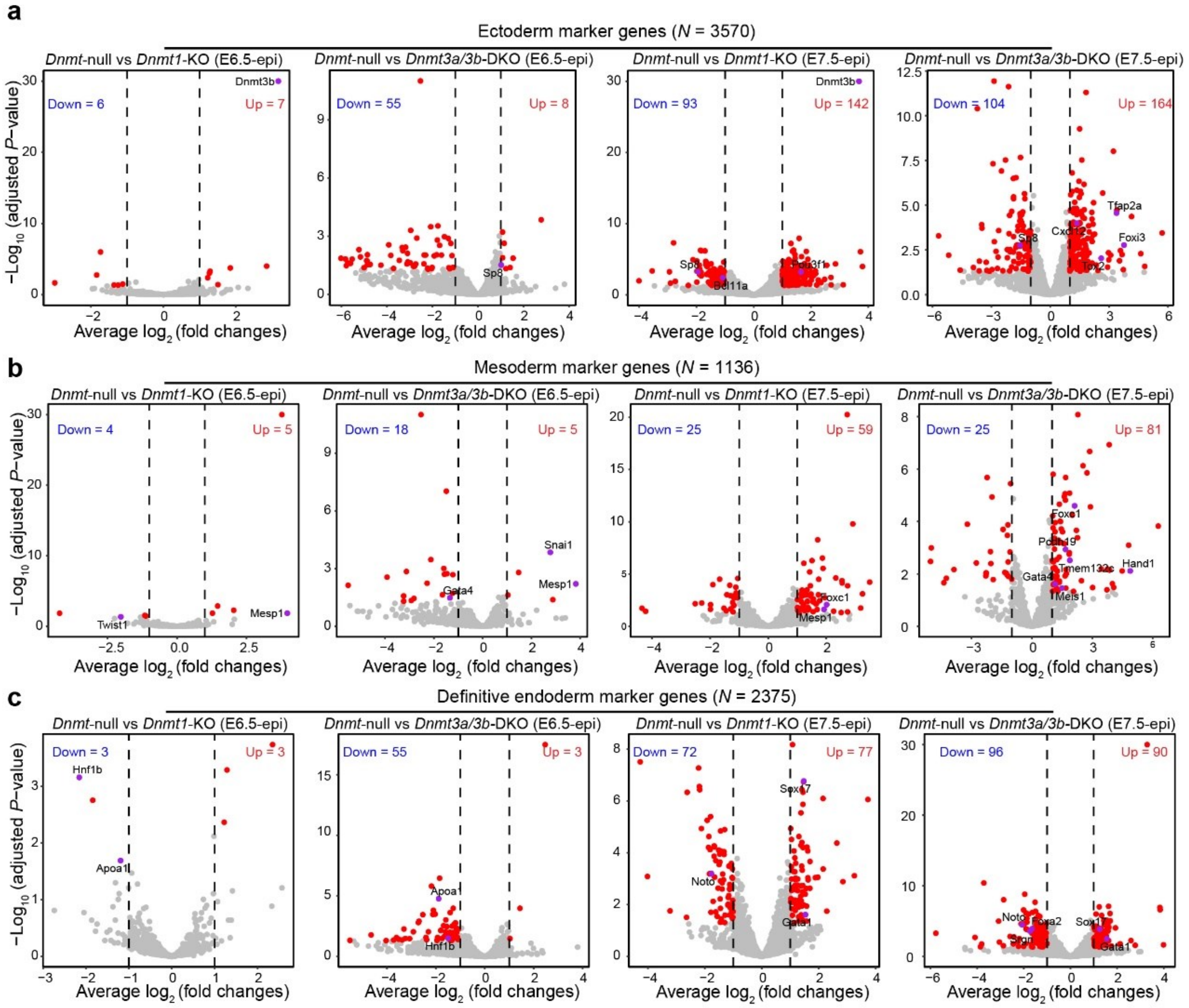


**Supplementary Fig. 7 | The mis-regulation of germ-layer enhancer-related genes in *Dnmt*-null embryos.**

**a-c,** Volcano plots show differentially expressed genes of *Dnmt*-null *vs Dnmt1*-KO and *Dnmt3a/3b*-DKO among genes located within 50kb around germ layer specific enhancer for ectoderm (**a**), mesoderm (**b**), and definitive endoderm (**c**), respectively, in E6.5 and E7.5 Epi (adjusted *P*-value < 0.05, FC > 2). *N*, the number of marker genes located close to germ layer specific enhancer (± 50kb) identified by Argelaguet et al[1]. *P*-values were calculated by Deseq2. All source data are provided as a Source Data file.

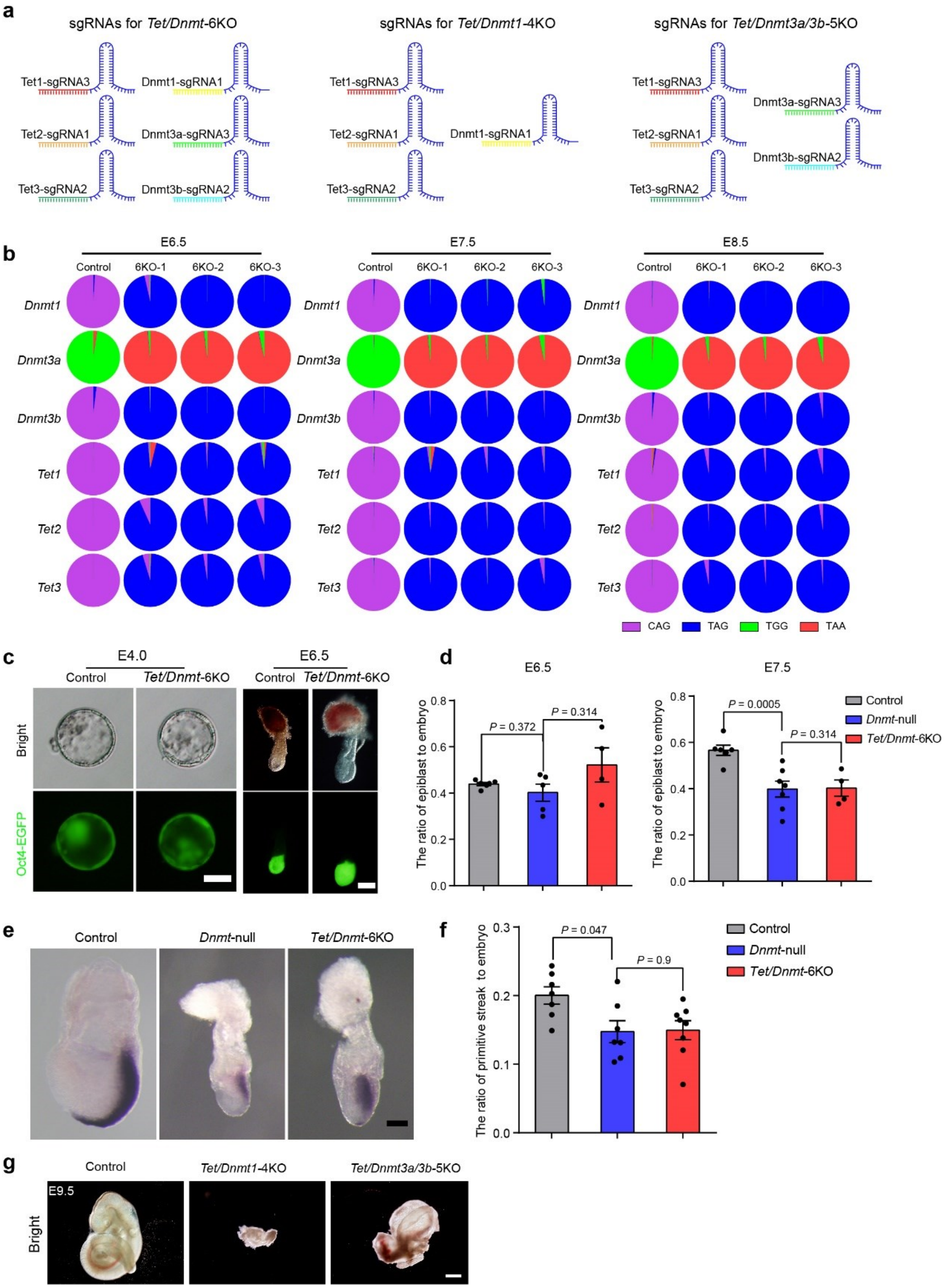
a
sgRNAs for Tet/Dnmt-6KO
sgRNAs for Tet/Dnmt1-4KO
sgRNAs for Tet/Dnmt3a/3b-5KO
Tet1-sgRNA3
Tet2-sgRNA1
Tet3-sgRNA2
Dnmt1-sgRNA1
Dnmt3a-sgRNA3
Dnmt3b-sgRNA2
b
E6.5
E7.5
E8.5
Control
6KO-1
6KO-2
6KO-3
Dnmt1
Dnmt3a
Dnmt3b
Tet1
Tet2
Tet3
CAG
TAG
TGG
TAA
c
E4.0
E6.5
Control
Tet/Dnmt-6KO
Bright
Oct4-EGFP
d
E6.5
E7.5
The ratio of epiblast to embryo
P = 0.372
P = 0.314
P = 0.0005
P = 0.314
Control
Dnmt-null
Tet/Dnmt-6KO
e
Control
Dnmt-null
Tet/Dnmt-6KO
f
The ratio of primitive streak to embryo
P = 0.047
P = 0.9
Control
Dnmt-null
Tet/Dnmt-6KO
g
Control
Tet/Dnmt1-4KO
Tet/Dnmt3a/3b-5KO
E9.5
Bright

**Supplementary Fig. 8 | IMGZ-mediated generation of *Dnmt*/*Tet* mutant embryos.** **a,** Schematic diagram of sgRNAs used to construct *Tet1/2/3*;*Dnmt1/3a/3b*-6KO, *Tet1/2/3*;*Dnmt1*-4KO, and *Tet1/2/3*;*Dnmt3a/3b*-5KO embryos through the IMGZ system. **b,** Deep sequencing analysis of genome mutant sites of one control and three *Tet/Dnmt*-6KO embryos at E6.5, E7.5, and E8.5. The results indicated the conversion of CAG or CGG codon of *Tet/Dnmt*-6KO into TAG or TAA stop codon in resultant embryos. **c,** Representative images of control and *Tet/Dnmt*-6KO embryos at E4.0 and E6.5. Green fluorescence indicates the expression of *Oct4-EGFP*. Three independent embryos were analyzed for each group. Scale bars, 50 μm in the left panel and 200 μm in the middle panel. **d,** The ratio of epiblast area to the whole embryo in control ($n$ = 6 for E6.5 and E7.5), *Dnmt*-null ($n$ = 5 for E6.5 and $n$ = 7 for E7.5), and *Tet/Dnmt*-6KO ($n$ = 4 for E6.5 and E7.5) embryos at E6.5 and E7.5. Epiblast was indicated by the signal of *Oct4-EGFP*. Data are mean ± s.e.m of indicated biological replicates *P*-values were determined by Student's unpaired two-sided *t*-test. **e,** Representative images of RNA in situ hybridization of *T* probe in the control, *Dnmt*-null, and *Tet/Dnmt*-6KO embryos at E7.5, showing primitive streak elongation failure in these embryos. More than three independent embryos were analyzed for each group. Scale bar, 100 μm. **f,** The ratio of primitive streak area to the whole embryo of control ($n$ = 7), *Dnmt*-null ($n$ = 7), and *Tet/Dnmt*-6KO ($n$ = 8) embryos at E7.5. The primitive streak was indicated by the signal of *T*. Data are mean ± s.e.m of indicated biological replicates. *P*-values were determined by Student's unpaired two-sided *t*-test. **g,** Representative images of control, *Tet/Dnmt1*-4KO, and *Tet/Dnmt3a/3b*-5KO embryos at E9.5 generated by the IMGZ system. Three independent embryos were analyzed for each group. Scale bar, 500 μm. All source data are provided as a Source Data file.

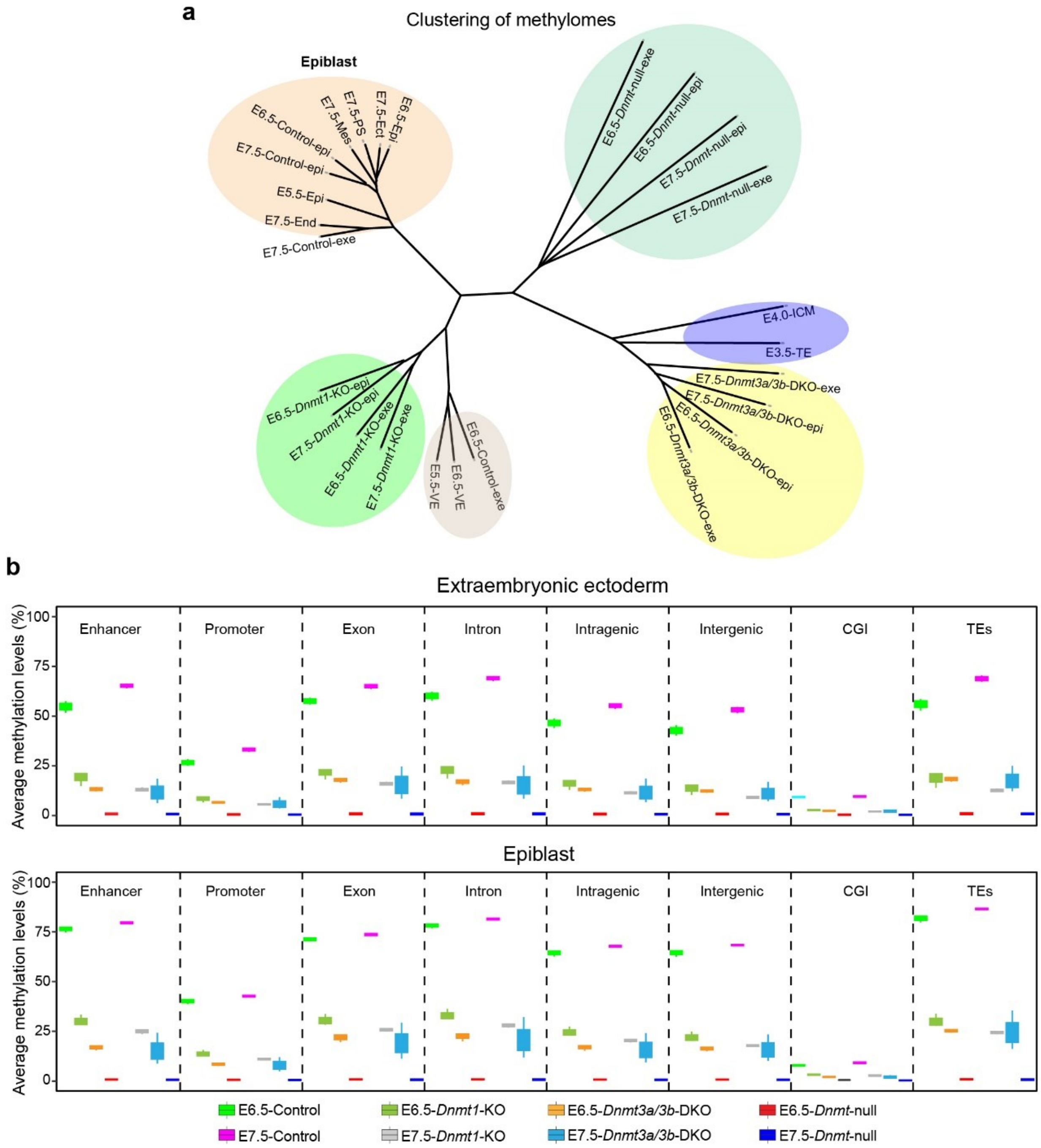


**Supplementary Fig. 9 | Methylome analysis of *Dnmt* mutant embryos.**

**a,** A dendrogram showing clustering of methylomes for Exe and Epi of four genotypes (control, *Dnmt1*-KO, *Dnmt3a/3b*-DKO, and *Dnmt*-null embryos) at E6.5 and E7.5. The methylome data of early embryos are produced by a previous study[2]. ICM, inner cell mass; TE, trophectoderm; VE, visceral endoderm; Ect, ectoderm; End, endoderm; Mes, mesoderm; PS, primitive streak. **b,** Box plots of CG methylation levels measured by WGBS in different annotated genomic elements in Exe and Epi of four genotypes at E6.5 and E7.5. The central lines are the median of data. The lower and upper hinges correspond to the 25th and 75th percentiles. The end of the lower and upper whiskers are 1.5 * IQR (inter-quartile range). Data beyond the end of the whiskers are plotted as outliers. All source data are provided as a Source Data file.

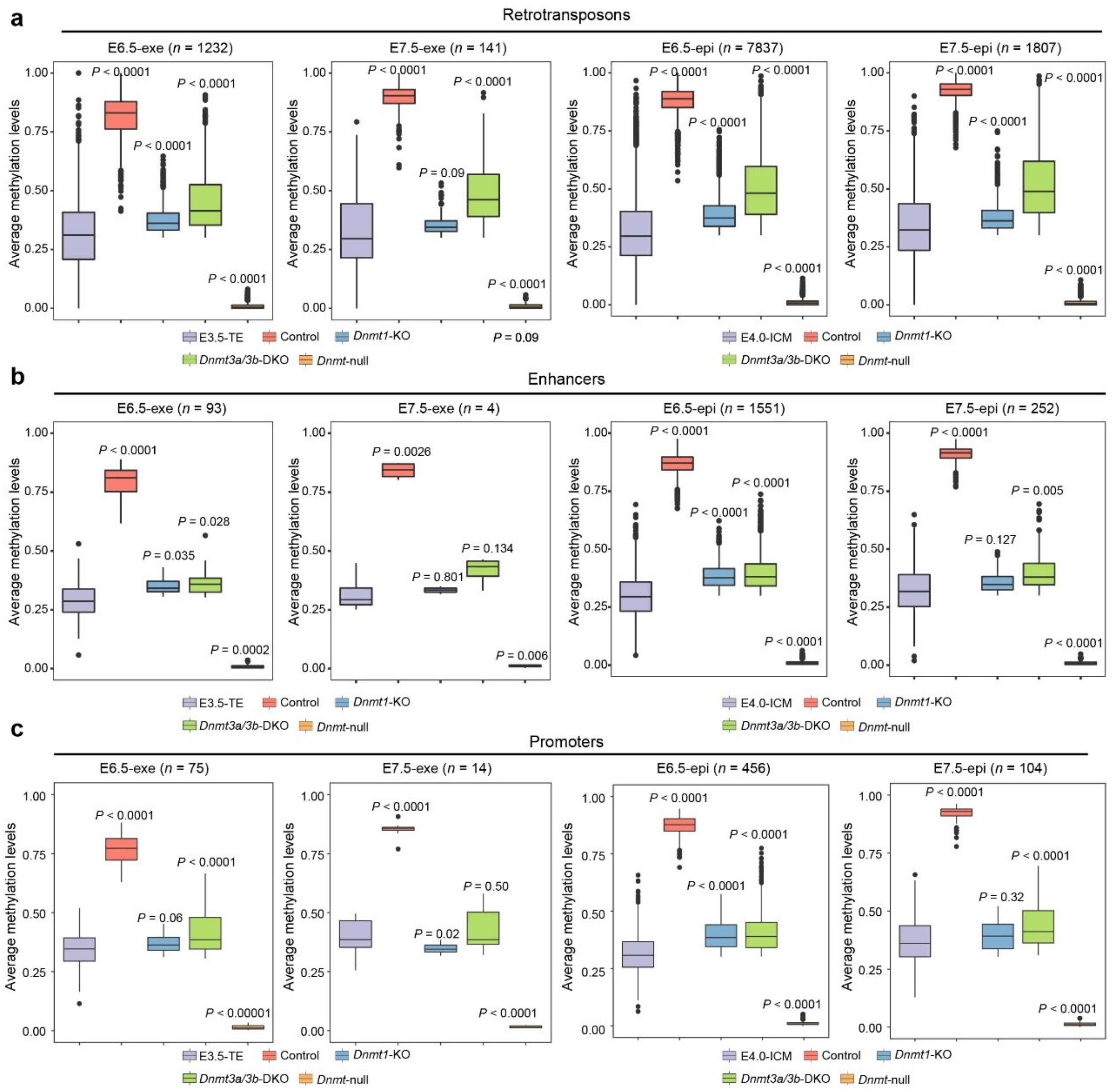


**Supplementary Fig. 10 | DNA methylation analysis of the overlapped hypermethylated elements maintained by DNMT1 and/or DNMT3A/3B.**

**a, b, c,** Box plots showed methylation levels of overlapped hypermethylated retrotransposons (**a**), enhancers (**b**), promoters (**c**) between *Dnmt1*-KO *vs* *Dnmt*-null and *Dnmt3a/3b*-DKO *vs* *Dnmt*-null in Exe and Epi at E6.5 and E7.5 related to Fig. 6b. E3.5 TE and E4.0 ICM data obtained from a previous study[2] show that DNA methylation has been deposited on these loci at the blastocyst stage. *n*, the number of overlapped elements. *P*-values were determined by Student's unpaired two-sided *t*-test. The central lines are the median of data. The lower and upper hinges correspond to the 25th and 75th percentiles. The end of the lower and upper whiskers are 1.5 * IQR (inter-quartile range). Data beyond the end of the whiskers are plotted as outliers. All source data are provided as a Source Data file.

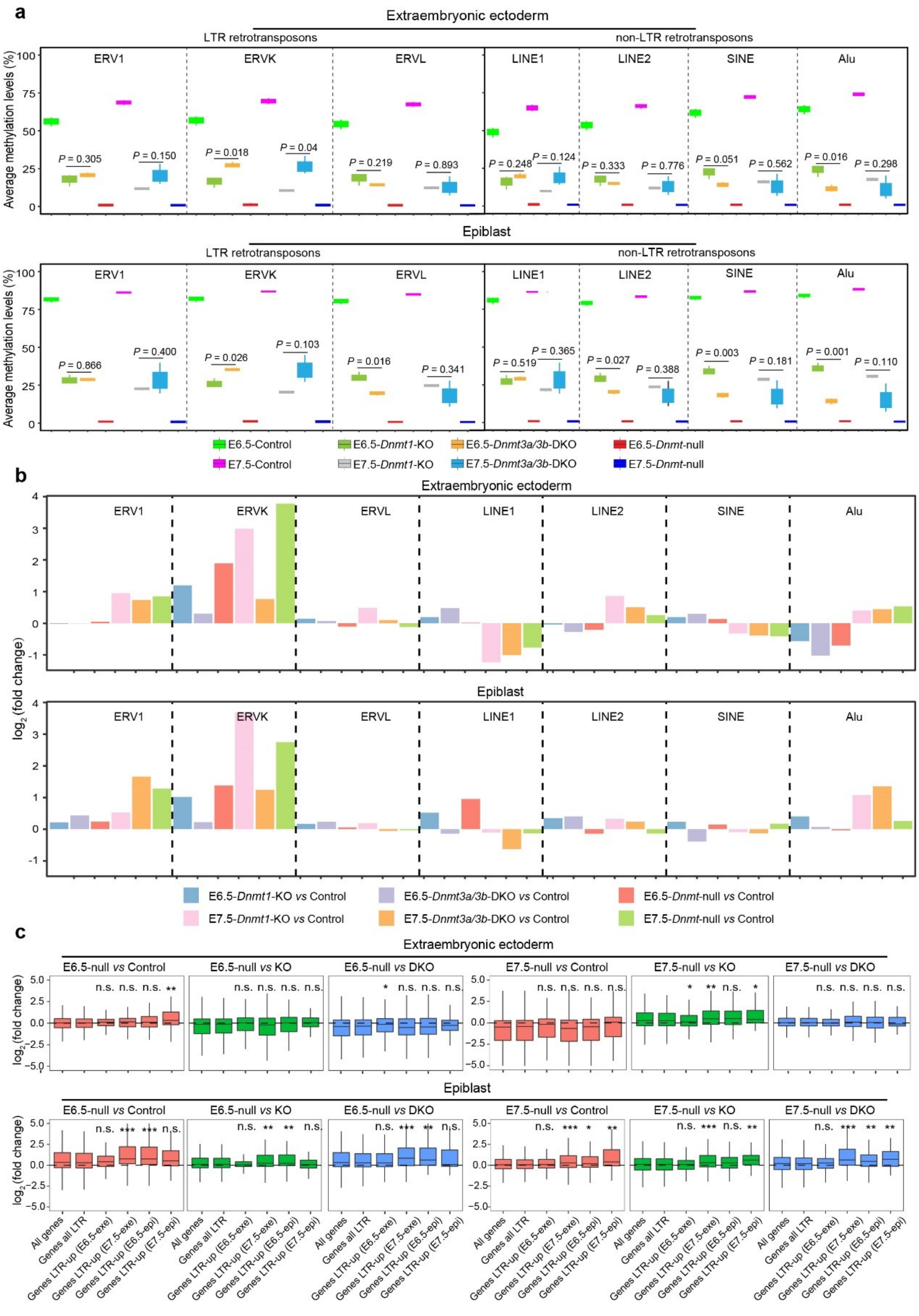
a
Extraembryonic ectoderm
LTR retrotransposons
non-LTR retrotransposons
ERV1
ERVK
ERVL
LINE1
LINE2
SINE
Alu
Average methylation levels (%)
P = 0.305
P = 0.150
P = 0.018
P = 0.04
P = 0.219
P = 0.893
P = 0.248
P = 0.124
P = 0.333
P = 0.776
P = 0.051
P = 0.562
P = 0.016
P = 0.298
Epiblast
LTR retrotransposons
non-LTR retrotransposons
ERV1
ERVK
ERVL
LINE1
LINE2
SINE
Alu
Average methylation levels (%)
P = 0.866
P = 0.400
P = 0.026
P = 0.103
P = 0.016
P = 0.341
P = 0.519
P = 0.365
P = 0.027
P = 0.388
P = 0.003
P = 0.181
P = 0.001
P = 0.110
E6.5-Control
E6.5-Dnmt1-KO
E6.5-Dnmt3a/3b-DKO
E6.5-Dnmt-null
E7.5-Control
E7.5-Dnmt1-KO
E7.5-Dnmt3a/3b-DKO
E7.5-Dnmt-null
b
Extraembryonic ectoderm
Epiblast
log2 (fold change)
ERV1
ERVK
ERVL
LINE1
LINE2
SINE
Alu
E6.5-Dnmt1-KO vs Control
E6.5-Dnmt3a/3b-DKO vs Control
E6.5-Dnmt-null vs Control
E7.5-Dnmt1-KO vs Control
E7.5-Dnmt3a/3b-DKO vs Control
E7.5-Dnmt-null vs Control
c
Extraembryonic ectoderm
E6.5-null vs Control
E6.5-null vs KO
E6.5-null vs DKO
E7.5-null vs Control
E7.5-null vs KO
E7.5-null vs DKO
log2 (fold change)
Epiblast
All genes
Genes all LTR
Genes LTR-up (E6.5-exe)
Genes LTR-up (E7.5-exe)
Genes LTR-up (E6.5-epi)
Genes LTR-up (E7.5-epi)
n.s.
*
**
***

**Supplementary Fig. 11 | Upregulation of retrotransposons and retrotransposon-related genes in *Dnmt* mutant embryos.**

**a,** Box plots show CG methylation levels of retrotransposons in Exe and Epi with different mutations at E6.5 and E7.5 measured by WGBS, including LTR retrotransposons (long terminal repeats) and non-LTR retrotransposons. The central lines are the median of data. The lower and upper hinges correspond to the 25th and 75th percentiles. The end of the lower and upper whiskers are 1.5 * IQR (inter-quartile range). Data beyond the end of the whiskers are plotted as outliers. *P*-values were determined by unpaired two-sided *t*-test. ERVs, endogenous retroviruses; LINEs, long interspersed elements; SINEs, short interspersed elements; the Alu sequences are named based on sharing a common cleavage site for the AluI restriction enzyme. **b,** Expression of retrotransposon families, including ERV1, ERVK, ERVL, LINE1, LINE2, SINE, and Alu, in Exe and Epi at E6.5 and E7.5 measured by RNA-seq, indicating that ERVK was significantly up-regulated in *Dnmt* mutant embryos (fold change > 2). **c,** Expression of all genes, genes located close to all LTRs (± 20kb), and genes located close to up-regulated LTRs in *Dnmt*-null (compared to both *Dnmt1*-KO and *Dnmt3a/3b*-DKO). The central lines are the median of data. The lower and upper hinges correspond to the 25th and 75th percentiles. The end of the lower and upper whiskers are 1.5 * IQR (inter-quartile range). Data beyond the end of the whiskers are plotted as outliers. *P*-values were calculated between the up-regulated LTR groups and All-LTR group by the two-sided Wilcoxon test. The exact *P*-values were listed in Source Data file. All source data are provided as a Source Data file.

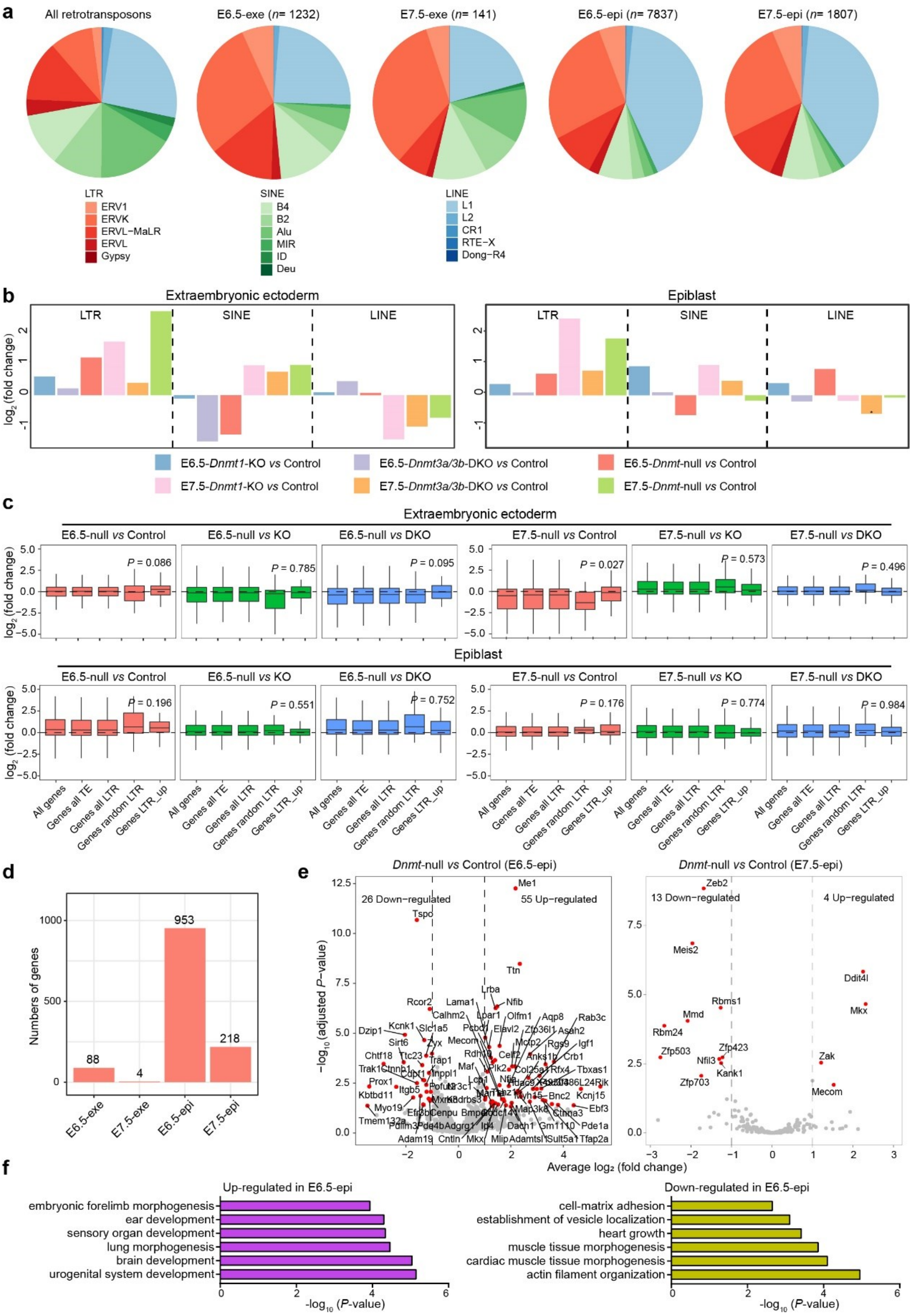

a
All retrotransposons
E6.5-exe (n= 1232)
E7.5-exe (n= 141)
E6.5-epi (n= 7837)
E7.5-epi (n= 1807)
LTR
ERV1
ERVK
ERVL−MaLR
ERVL
Gypsy
SINE
B4
B2
Alu
MIR
ID
Deu
LINE
L1
L2
CR1
RTE−X
Dong−R4
b
Extraembryonic ectoderm
Epiblast
LTR
SINE
LINE
log2 (fold change)
E6.5-Dnmt1-KO vs Control
E6.5-Dnmt3a/3b-DKO vs Control
E6.5-Dnmt-null vs Control
E7.5-Dnmt1-KO vs Control
E7.5-Dnmt3a/3b-DKO vs Control
E7.5-Dnmt-null vs Control
c
Extraembryonic ectoderm
E6.5-null vs Control
E6.5-null vs KO
E6.5-null vs DKO
E7.5-null vs Control
E7.5-null vs KO
E7.5-null vs DKO
P = 0.086
P = 0.785
P = 0.095
P = 0.027
P = 0.573
P = 0.496
Epiblast
P = 0.196
P = 0.551
P = 0.752
P = 0.176
P = 0.774
P = 0.984
log2 (fold change)
All genes
Genes all TE
Genes all LTR
Genes random LTR
Genes LTR_up
d
Numbers of genes
88
4
953
218
E6.5-exe
E7.5-exe
E6.5-epi
E7.5-epi
e
Dnmt-null vs Control (E6.5-epi)
Dnmt-null vs Control (E7.5-epi)
26 Down-regulated
55 Up-regulated
13 Down-regulated
4 Up-regulated
−log10 (adjusted P−value)
Average log2 (fold change)
Me1
Tspo
Ttn
Zeb2
Meis2
Ddit4l
Mkx
Rbms1
Mmd
Rbm24
Zfp503
Zfp423
Nfil3
Kank1
Zfp703
Zak
Mecom
f
Up-regulated in E6.5-epi
embryonic forelimb morphogenesis
ear development
sensory organ development
lung morphogenesis
brain development
urogenital system development
Down-regulated in E6.5-epi
cell-matrix adhesion
establishment of vesicle localization
heart growth
muscle tissue morphogenesis
cardiac muscle tissue morphogenesis
actin filament organization
-log10 (P-value)

**Supplementary Fig. 12 | Slight influence of overlapped methylated retrotransposons and enhancers on gene expression in *Dnmt*-null embryos.**

**a,** Pie charts show the distribution of retrotransposon families in overlapped hypermethylated retrotransposons of Exe and Epi at E6.5 and E7.5 related to Fig. 6b. All retrotransposons show the distribution of retrotransposon families in the mouse genome. **b,** Expression of overlapped methylated retrotransposons (class LTR, SINE, and LINE) in Exe and Epi at E6.5 and E7.5, indicating that overlapped LTRs were upregulated in *Dnmt* mutant embryos. **c,** Expression of all genes, genes located close to all TEs, genes located to all LTRs, genes located to random LTRs ($n = 50$), and genes located close to all up-regulated LTRs with DNA hypomethylation in *Dnmt*-null (compared to *Dnmt1*-KO and *Dnmt3a/3b*-DKO) in Exe and Epi at E6.5 and E7.5. The central lines are the median of data. The lower and upper hinges correspond to the 25th and 75th percentiles. The end of the lower and upper whiskers are 1.5 * IQR (inter-quartile range). Data beyond the end of the whiskers are plotted as outliers. *P*-values were calculated between LTR-up and All-LTR by the two-sided Wilcoxon test. **d,** Bar plots show the numbers of genes regulated by the overlapped hypermethylated enhancers (HMEs) in Exe and Epi at E6.5 and E7.5 related to Fig. 6b. **e,** Volcano plots show the DEGs (fold change > 2 and adjusted *P*-value < 0.05) in HME-regulated genes in E6.5 and E7.5 Epi. *P*-values were calculated by Deseq2. **f,** Gene Ontology enrichments of DEGs in HME-regulated genes in E6.5 Epi related to (**e**). *P*-values were calculated by Metascape. All source data are provided as a Source Data file.

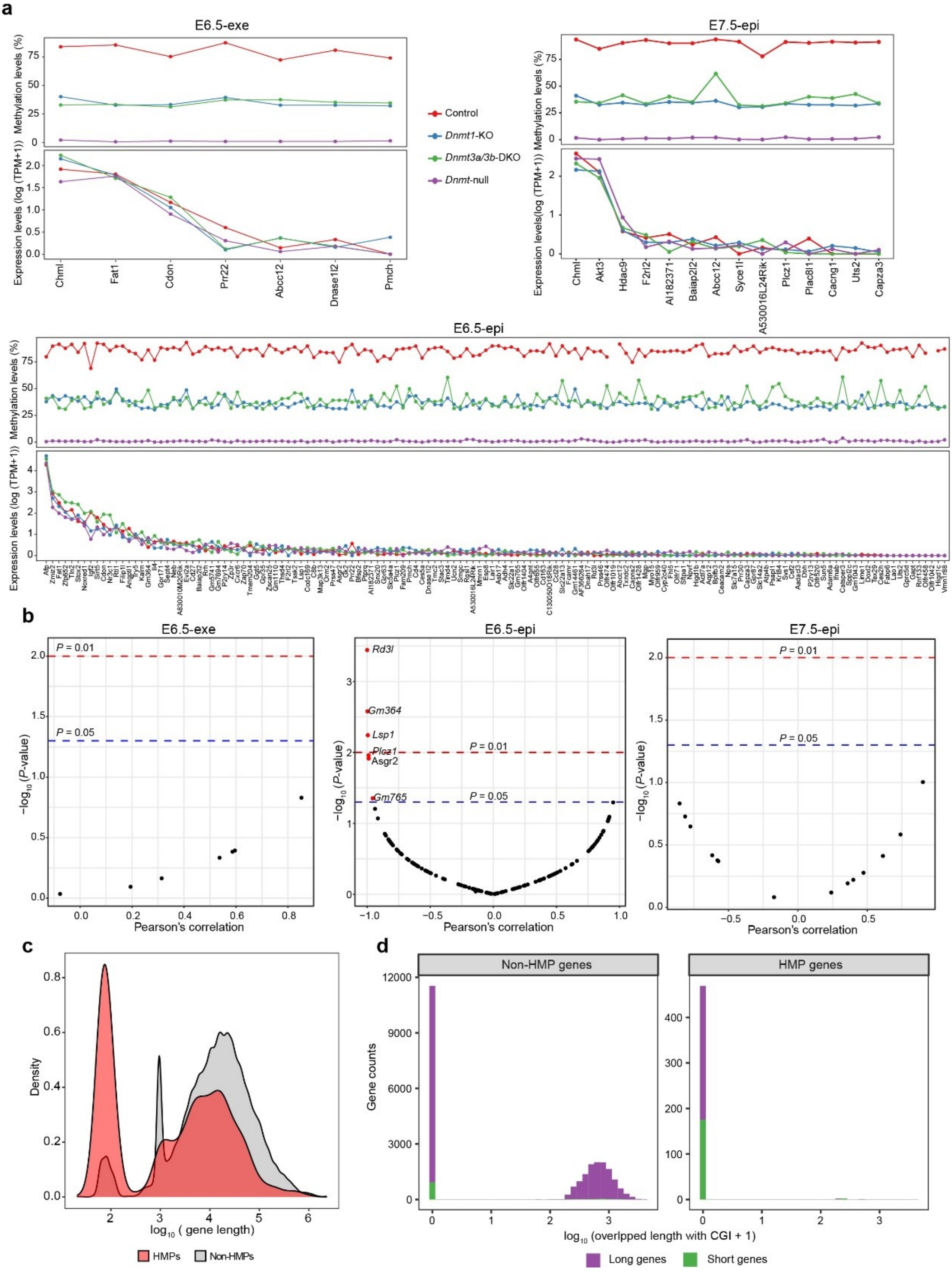
a
E6.5-exe
Methylation levels (%)
Expression levels (log (TPM+1))
Chml
Fat1
Cdon
Prr22
Abcc12
Dnase1l2
Pmch
Control
Dnmt1-KO
Dnmt3a/3b-DKO
Dnmt-null
E7.5-epi
Methylation levels (%)
Expression levels(log (TPM+1))
Chml
Akt3
Hdac9
F2rl2
AI182371
Baiap2l2
Abcc12
Syce1l
A530016L24Rik
Plcz1
Plac8l1
Cacng1
Uts2
Capza3
E6.5-epi
Methylation levels (%)
Expression levels (log (TPM+1))
b
E6.5-exe
P = 0.01
P = 0.05
−log10 (P-value)
Pearson's correlation
E6.5-epi
Rd3l
Gm364
Lsp1
Plcz1
Asgr2
Gm765
P = 0.01
P = 0.05
−log10 (P-value)
Pearson's correlation
E7.5-epi
P = 0.01
P = 0.05
−log10 (P-value)
Pearson's correlation
c
Density
log10 ( gene length)
HMPs
Non-HMPs
d
Non-HMP genes
HMP genes
Gene counts
log10 (overlpped length with CGI + 1)
Long genes
Short genes

**Supplementary Fig. 13 | Analysis of overlapped HMP-related genes.**

**a,** Scatter diagrams show the DNA methylation level and RNA expression level of the protein coding genes related to HMPs in Exe and Epi of four genotypes at E6.5 and E7.5 (no overlapped protein coding genes related to HMPs identified in E7.5 Exe). **b,** Pearson's correlation of DNA methylation and RNA expression of the protein coding genes related to (**a**), indicating that the expression of protein coding genes related to HMPs is not influence by DNA methylation. *P*-value was determined by two-sided R function 'cor. test' which is based on Pearson's coefficient and follows in a *t* distribution. **c,** Density plots show the distribution of gene length of the HMP-related genes and non-HMP-related genes. HMPs contain all the overlapped HMPs shown in Fig. 6b; Non-HMPs refer to other gene promotes besides the HMPs. **d,** Histograms show the distribution of covered CGI length in HMP-related genes and non-HMP-related genes, indicating lack of CGI regions in the promoter of the overlapped HMP-related genes. All source data are provided as a Source Data file.

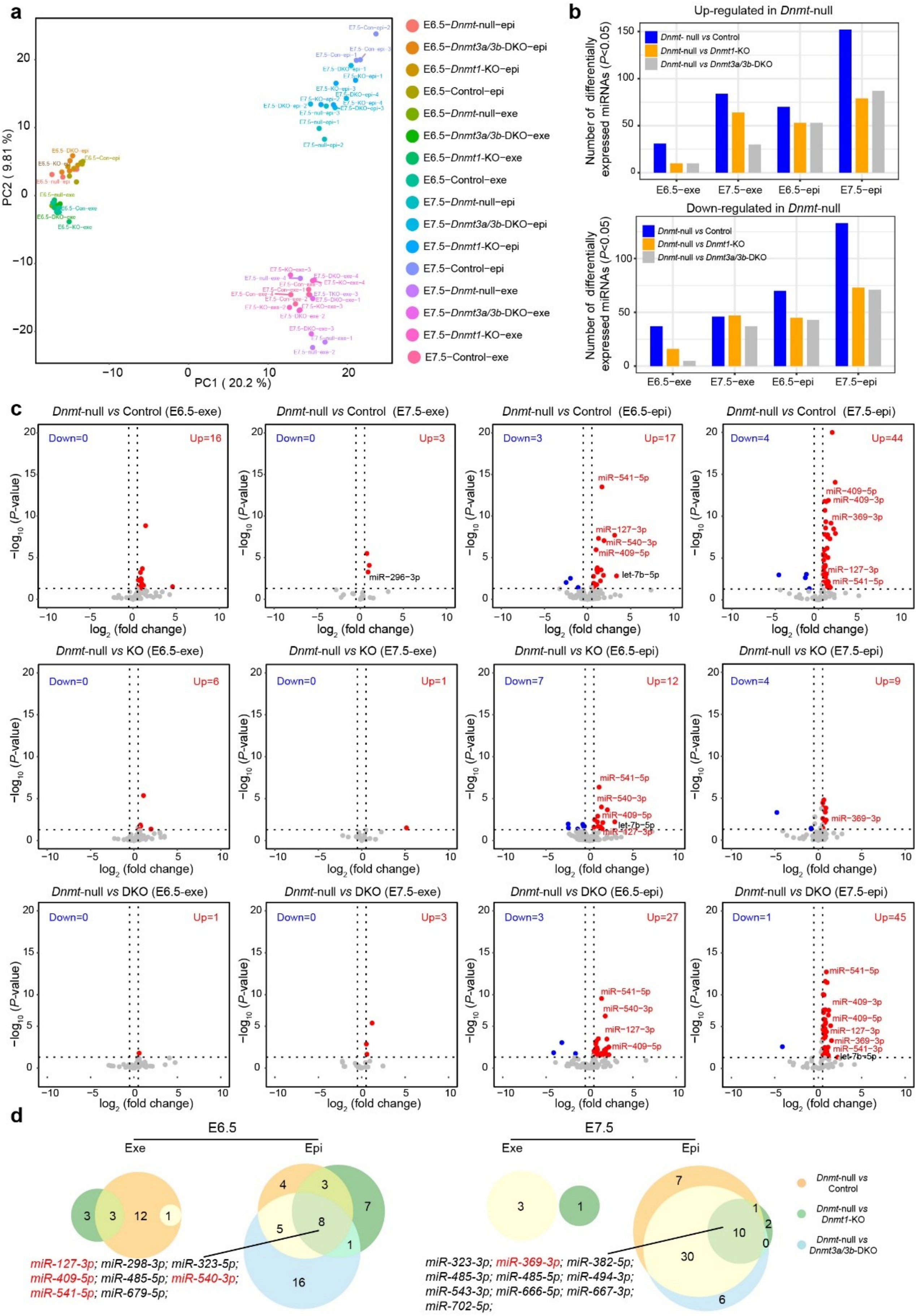
a
PC1 ( 20.2 %)
PC2 ( 9.81 %)
E6.5−Dnmt-null−epi
E6.5−Dnmt3a/3b-DKO−epi
E6.5−Dnmt1-KO−epi
E6.5−Control−epi
E6.5−Dnmt-null−exe
E6.5−Dnmt3a/3b-DKO−exe
E6.5−Dnmt1-KO−exe
E6.5−Control−exe
E7.5−Dnmt-null−epi
E7.5−Dnmt3a/3b-DKO−epi
E7.5−Dnmt1-KO−epi
E7.5−Control−epi
E7.5−Dnmt-null−exe
E7.5−Dnmt3a/3b-DKO−exe
E7.5−Dnmt1-KO−exe
E7.5−Control−exe
b
Up-regulated in Dnmt-null
Down-regulated in Dnmt-null
Number of differentially expressed miRNAs (P<0.05)
Dnmt- null vs Control
Dnmt-null vs Dnmt1-KO
Dnmt-null vs Dnmt3a/3b-DKO
E6.5−exe
E7.5−exe
E6.5−epi
E7.5−epi
c
Dnmt-null vs Control (E6.5-exe)
Dnmt-null vs Control (E7.5-exe)
Dnmt-null vs Control (E6.5-epi)
Dnmt-null vs Control (E7.5-epi)
Dnmt-null vs KO (E6.5-exe)
Dnmt-null vs KO (E7.5-exe)
Dnmt-null vs KO (E6.5-epi)
Dnmt-null vs KO (E7.5-epi)
Dnmt-null vs DKO (E6.5-exe)
Dnmt-null vs DKO (E7.5-exe)
Dnmt-null vs DKO (E6.5-epi)
Dnmt-null vs DKO (E7.5-epi)
Down=0 Up=16
Down=0 Up=3
Down=3 Up=17
Down=4 Up=44
Down=0 Up=6
Down=0 Up=1
Down=7 Up=12
Down=4 Up=9
Down=0 Up=1
Down=0 Up=3
Down=3 Up=27
Down=1 Up=45
−log10 (P-value)
log2 (fold change)
miR−296−3p
miR−541−5p
miR−127−3p
miR−540−3p
miR−409−5p
let−7b−5p
miR−409−3p
miR−369−3p
miR−541−3p
d
E6.5
E7.5
Exe
Epi
miR-127-3p; miR-298-3p; miR-323-5p;
miR-409-5p; miR-485-5p; miR-540-3p;
miR-541-5p; miR-679-5p;
miR-323-3p; miR-369-3p; miR-382-5p;
miR-485-3p; miR-485-5p; miR-494-3p;
miR-543-3p; miR-666-5p; miR-667-3p;
miR-702-5p;
Dnmt-null vs Control
Dnmt-null vs Dnmt1-KO
Dnmt-null vs Dnmt3a/3b-DKO

**Supplementary Fig. 14 | HMP-related miRNAs are up-regulated in *Dnmt*-null embryos.**

**a,** PCA analysis of miRNA profiling of Exe and Epi from four groups (control, *Dnmt1*-KO, *Dnmt3a/3b*-DKO, and *Dnmt*-null) at E6.5 and E7.5. **b,** Bar plots represent the number of significantly up-regulated and down-regulated miRNAs in *Dnmt*-null embryos, compared to control, *Dnmt1*-KO, and *Dnmt3a/3b*-DKO, respectively. *P*-values were calculated by Deseq2. **c,** Volcano plots show the differently expressed miRNAs related to overlapped HMPs, including *Dnmt*-null compared to control, *Dnmt1*-KO, and *Dnmt3a/3b*-DKO. *let-7b-3p* and *miR-296-3p* highlighted in black are not located at the *Dlk1-Dio3* locus. *P*-values were calculated by Deseq2; $Log_2$ (fold change) > 0.5; *P*-value < 0.05. **d,** Veen diagrams show overlapped differentially expressed miRNAs related to HMPs shown in Fig. 7a among *Dnmt*-null *vs* control, *Dnmt*-null *vs* *Dnmt1*-KO, and *Dnmt*-null *vs* *Dnmt3a/3b*-DKO in Exe and Epi at E6.5 and E7.5. Overlapped miRNAs are shown below and the miRNAs highlighted in red are deleted for the functional analysis below. All source data are provided as a Source Data file.

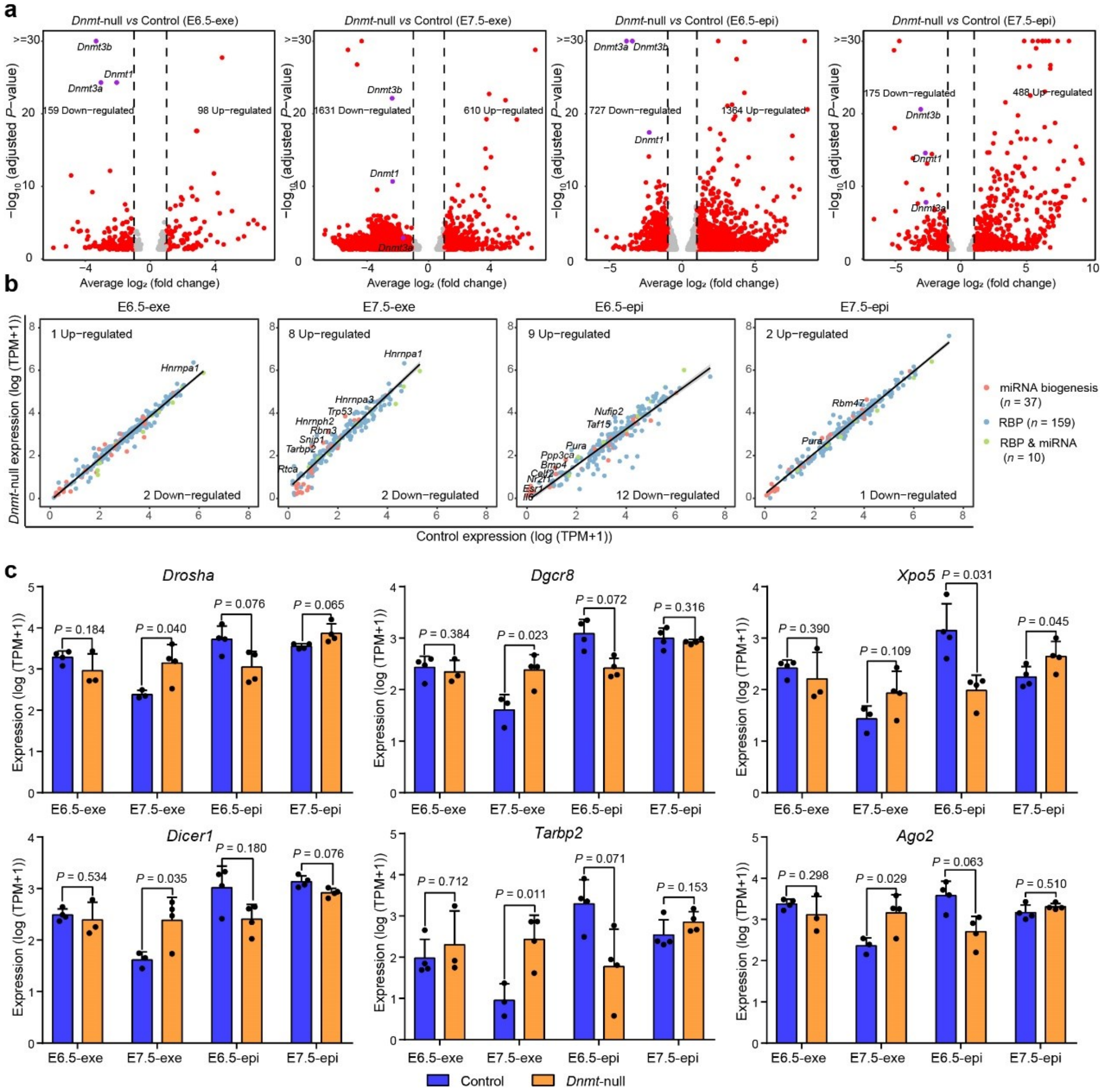


**Supplementary Fig. 15 | miRNAs biogenesis-related pathways are not affected in *Dnmt*-null embryos.**

**a,** Volcano plots show differentially expressed genes between *Dnmt*-null and control in Exe and Epi at E6.5 and E7.5 (adjusted *P*-value < 0.05, FC > 2). *P*-values were calculated by Deseq2. **b,** Plots show the expression of genes encoding miRNA biogenesis-related proteins (*n* = 37, from GO0035196 in MGI), RNA-binding proteins (RBP, *n* = 159, identified by Treiber et al.[3]), and both (*n* = 10) in control and *Dnmt*-null embryos (adjusted *P*-value < 0.05, FC > 2). A few genes were up-regulated in *Dnmt*-null embryos, majority of which are RNA binding proteins that may be not critical for miRNA biogenesis. **c,** Expression of key genes related to miRNA biogenesis in control and *Dnmt*-null embryos. Data are mean ± s.e.m of four biological replicates. *P*-values were determined by Student's unpaired two-sided *t*-test. All source data are provided as a Source Data file.

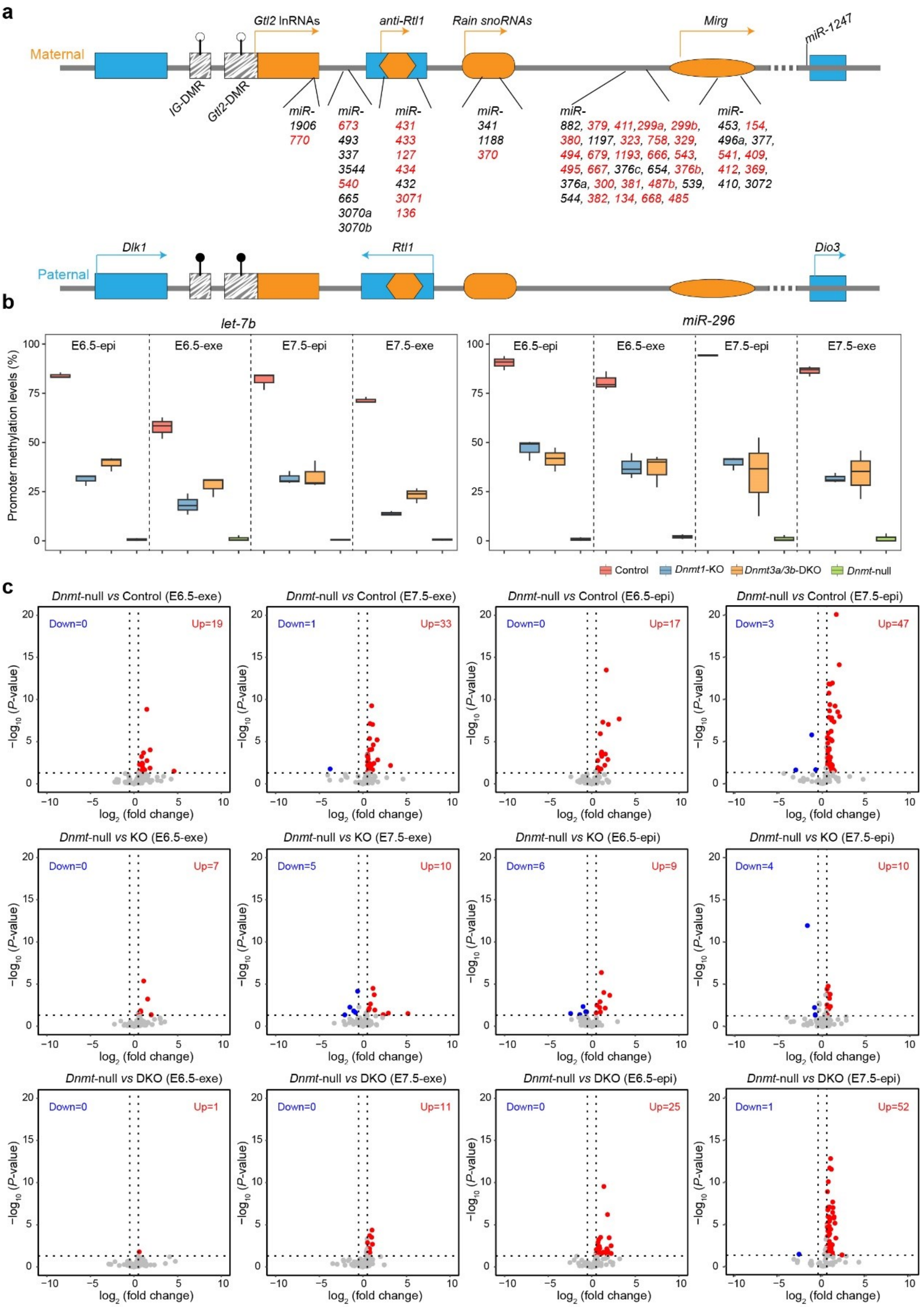
a
Maternal
Paternal
IG-DMR
Gtl2-DMR
Gtl2 lnRNAs
anti-Rtl1
Rain snoRNAs
Mirg
miR-1247
Dlk1
Rtl1
Dio3
miR- 1906 770
miR- 673 493 337 3544 540 665 3070a 3070b
miR- 431 433 127 434 432 3071 136
miR- 341 1188 370
miR- 882, 379, 411, 299a, 299b, 380, 1197, 323, 758, 329, 494, 679, 1193, 666, 543, 495, 667, 376c, 654, 376b, 376a, 300, 381, 487b, 539, 544, 382, 134, 668, 485
miR- 453, 154, 496a, 377, 541, 409, 412, 369, 410, 3072
b
let-7b
miR-296
E6.5-epi
E6.5-exe
E7.5-epi
E7.5-exe
Promoter methylation levels (%)
Control
Dnmt1-KO
Dnmt3a/3b-DKO
Dnmt-null
c
Dnmt-null vs Control (E6.5-exe)
Dnmt-null vs Control (E7.5-exe)
Dnmt-null vs Control (E6.5-epi)
Dnmt-null vs Control (E7.5-epi)
Dnmt-null vs KO (E6.5-exe)
Dnmt-null vs KO (E7.5-exe)
Dnmt-null vs KO (E6.5-epi)
Dnmt-null vs KO (E7.5-epi)
Dnmt-null vs DKO (E6.5-exe)
Dnmt-null vs DKO (E7.5-exe)
Dnmt-null vs DKO (E6.5-epi)
Dnmt-null vs DKO (E7.5-epi)
Down=0 Up=19
Down=1 Up=33
Down=0 Up=17
Down=3 Up=47
Down=0 Up=7
Down=5 Up=10
Down=6 Up=9
Down=4 Up=10
Down=0 Up=1
Down=0 Up=11
Down=0 Up=25
Down=1 Up=52
−log10 (P-value)
log2 (fold change)

**Supplementary Fig. 16 | The majority of up-regulated HMP-related miRNAs in *Dnmt*-null embryos are located at the *Dlk1-Dio3* imprinting region.**

**a,** Schematic illustration of *Dlk1-Dio3* miRNAs on mouse chromosome 12. The miRNAs highlighted in red were up-regulated in *Dnmt*-null embryos compared to control. **b,** Box plots show CG methylation levels of promoters (TSS ± 2kb) of *let-7b* and *miR-296* in different mutant embryos at E6.5 and E7.5 measured by WGBS. The central lines are the median of data. The lower and upper hinges correspond to the 25th and 75th percentiles. The end of the lower and upper whiskers are 1.5 * IQR (inter-quartile range). Data beyond the end of the whiskers are plotted as outliers. **c,** Volcano plots show the differently expressed *Dlk1-Dio3* miRNA in *Dnmt*-null compared to control, *Dnmt1*-KO, and *Dnmt3a/3b*-DKO. *P*-values were calculated by Deseq2; $Log_2$ (fold change) > 0.5; *P*-value < 0.05. All source data are provided as a Source Data file.

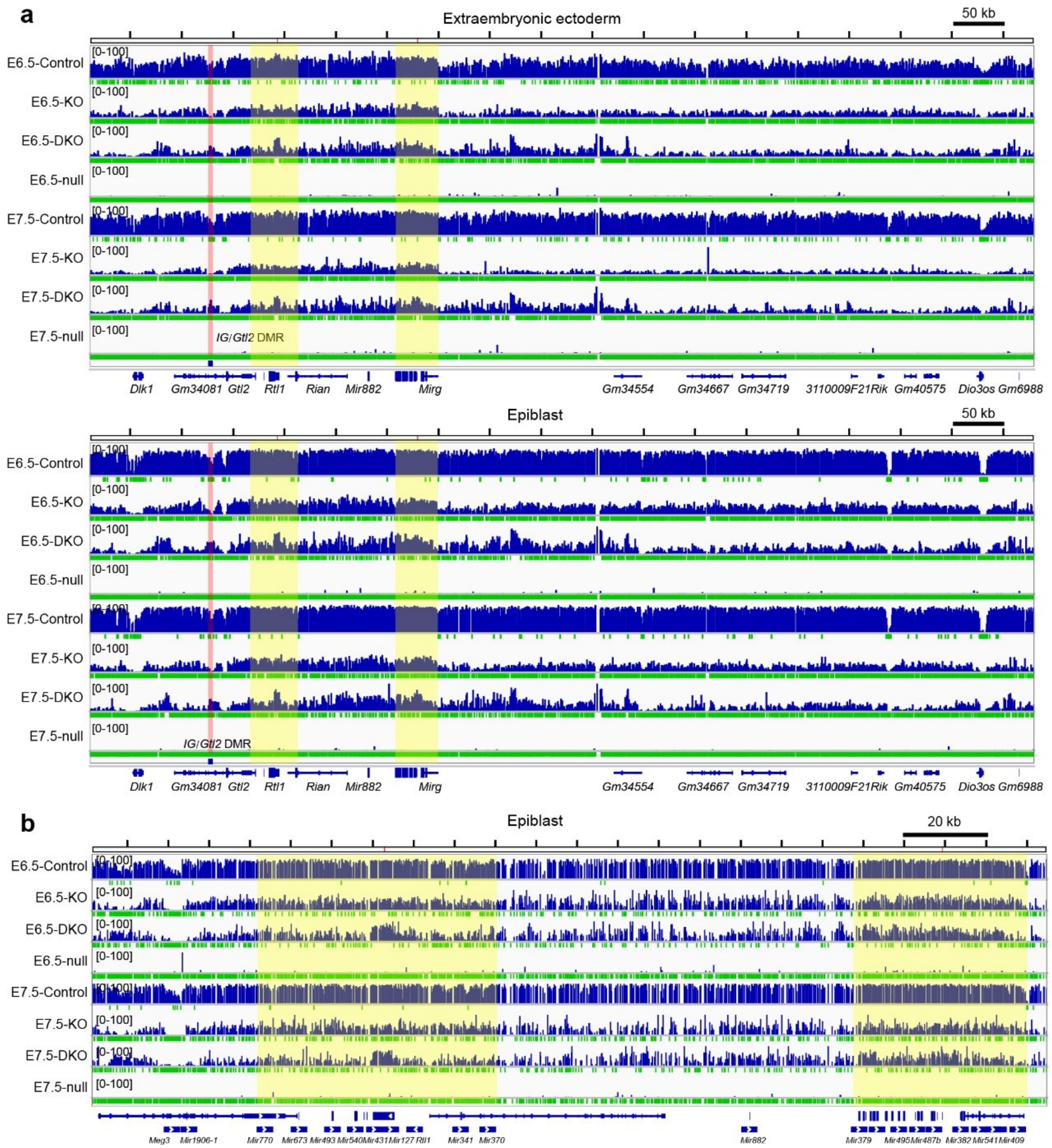
a
Extraembryonic ectoderm
50 kb
E6.5-Control
E6.5-KO
E6.5-DKO
E6.5-null
E7.5-Control
E7.5-KO
E7.5-DKO
E7.5-null
[0-100]
IG/Gtl2 DMR
Dlk1 Gm34081 Gtl2 Rtl1 Rian Mir882 Mirg Gm34554 Gm34667 Gm34719 3110009F21Rik Gm40575 Dio3os Gm6988
Epiblast
50 kb
b
Epiblast
20 kb
Meg3 Mir1906-1 Mir770 Mir673 Mir493 Mir540 Mir431 Mir127 Rtl1 Mir341 Mir370 Mir882 Mir379 Mir495 Mir487b Mir382 Mir541 Mir409

**Supplementary Fig. 17 | Methylation state of the *Dlk1-Dio3* locus in *Dnmt* mutant embryos.**

**a,** Representative genome browser snapshots of *Dlk1-Dio3* methylation profiles for Exe and Epi of four groups (control, *Dnmt1*-KO, *Dnmt3a/3b*-DKO, and *Dnmt*-null embryos) at E6.5 and E7.5. The red-shaded box indicates the *IG/Gtl2* DMR (differentially DNA-methylated region). The yellow-shaded box indicates the promoter regions containing many miRNAs. Green bars below the horizontal line denote the cytosine detected as unmethylated to discriminate from undetected cytosine sites. Resolution, 50kb. **b,** Representative genome browser snapshots of promoter methylation profiles for Epi of four groups at E6.5 and E7.5. The yellow-shaded box indicates the promoter regions containing many miRNAs. Resolution, 20kb. All source data are provided as a Source Data file.

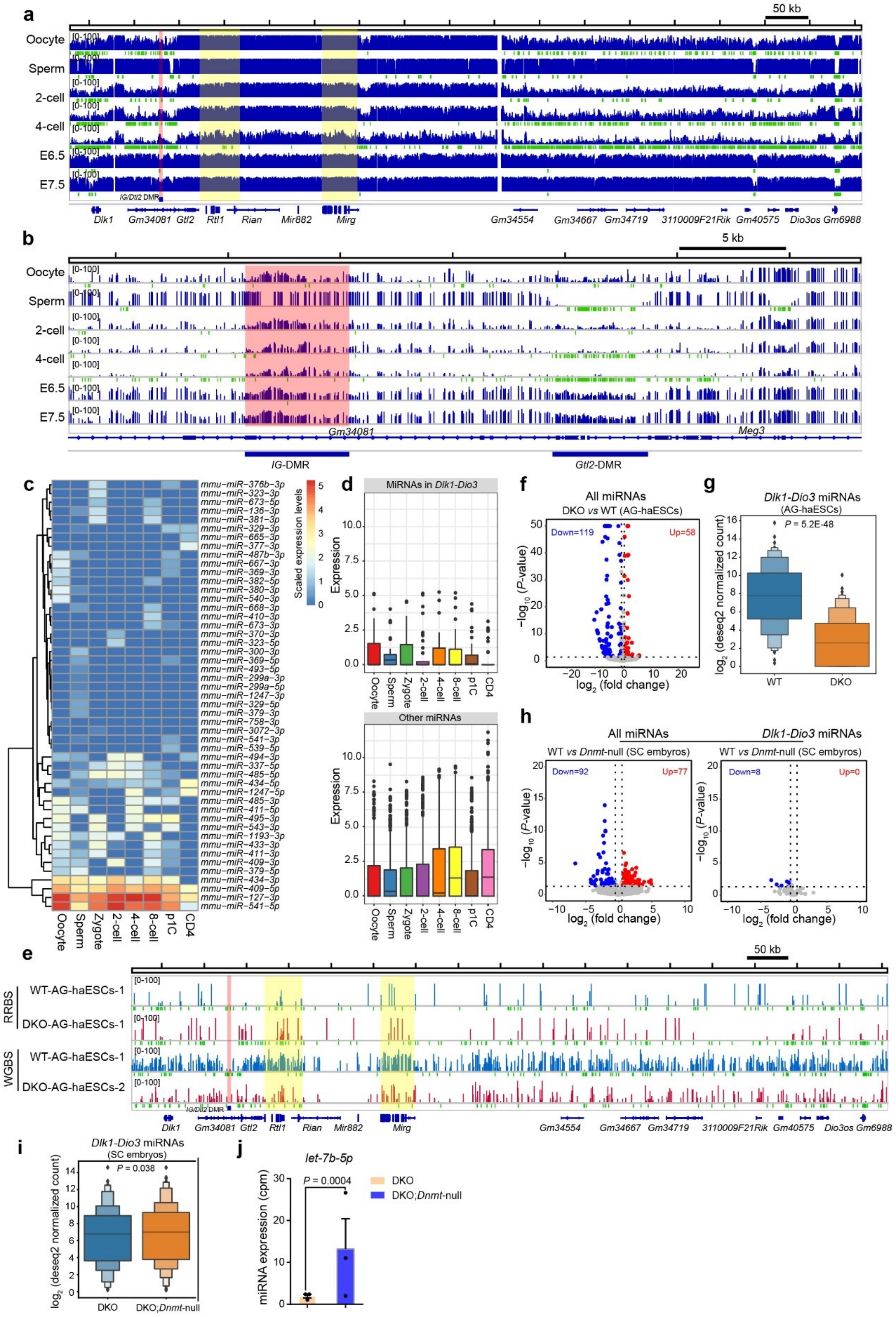
a
50 kb
Oocyte
Sperm
2-cell
4-cell
E6.5
E7.5
[0-100]
IG/Dtl2 DMR
Dlk1
Gm34081
Gtl2
Rtl1
Rian
Mir882
Mirg
Gm34554
Gm34667
Gm34719
3110009F21Rik
Gm40575
Dio3os
Gm6988
b
5 kb
Gm34081
Meg3
IG-DMR
Gtl2-DMR
c
Scaled expression levels
mmu−miR−376b−3p
mmu−miR−323−3p
mmu−miR−673−5p
mmu−miR−136−3p
mmu−miR−381−3p
mmu−miR−329−3p
mmu−miR−665−3p
mmu−miR−377−3p
mmu−miR−487b−3p
mmu−miR−667−3p
mmu−miR−369−3p
mmu−miR−382−5p
mmu−miR−380−3p
mmu−miR−540−3p
mmu−miR−668−3p
mmu−miR−410−3p
mmu−miR−673−3p
mmu−miR−370−3p
mmu−miR−323−5p
mmu−miR−300−3p
mmu−miR−369−5p
mmu−miR−493−5p
mmu−miR−299a−3p
mmu−miR−299a−5p
mmu−miR−1247−3p
mmu−miR−329−5p
mmu−miR−379−3p
mmu−miR−758−3p
mmu−miR−3072−3p
mmu−miR−541−3p
mmu−miR−539−5p
mmu−miR−494−3p
mmu−miR−337−5p
mmu−miR−485−5p
mmu−miR−434−5p
mmu−miR−1247−5p
mmu−miR−485−3p
mmu−miR−411−5p
mmu−miR−495−3p
mmu−miR−543−3p
mmu−miR−1193−3p
mmu−miR−433−3p
mmu−miR−411−3p
mmu−miR−409−3p
mmu−miR−379−5p
mmu−miR−434−3p
mmu−miR−409−5p
mmu−miR−127−3p
mmu−miR−541−5p
Oocyte Sperm Zygote 2-cell 4-cell 8-cell p1C CD4
d
MiRNAs in Dlk1-Dio3
Other miRNAs
Expression
e
RRBS
WGBS
WT-AG-haESCs-1
DKO-AG-haESCs-1
WT-AG-haESCs-1
DKO-AG-haESCs-2
f
All miRNAs
DKO vs WT (AG-haESCs)
Down=119
Up=58
−log10 (P-value)
log2 (fold change)
g
Dlk1-Dio3 miRNAs
(AG-haESCs)
P = 5.2E-48
log2 (deseq2 normalized count)
WT
DKO
h
All miRNAs
Dlk1-Dio3 miRNAs
WT vs Dnmt-null (SC embryos)
Down=92
Up=77
Down=8
Up=0
i
Dlk1-Dio3 miRNAs
(SC embryos)
P = 0.038
DKO
DKO;Dnmt-null
j
let-7b-5p
P = 0.0004
miRNA expression (cpm)
DKO
DKO;Dnmt-null

**Supplementary Fig. 18 | *Dlk1-Dio3* miRNAs are suppressed in pre-implantation embryos and DKO-AG-haESCs.**

**a,** Genome browser view of *Dlk1-Dio3* methylation profiles in gametes and early embryos (GSE56697[4]). The red-shaded box indicates the *IG/Gtl2* DMRs. The yellow-shaded box indicates the promoter regions containing miRNAs. Green bars below the horizontal line denote the cytosine detected as unmethylated to discriminate from undetected cytosine sites. Resolution, 50kb. **b,** Genome browser view of *IG*-DMR and *Gtl2*-DMR methylation profiles. The red-shaded box indicates the *IG*-DMR. Resolution, 5kb. **c,** Heatmap shows the expression of *Dlk1-Dio3* miRNAs in gametes and early embryos (SRP045287[5]). p1C, parthenogenetic one-cell embryos. **d,** Bar plots show the expression of *Dlk1-Dio3* miRNAs and other miRNAs in gametes and pre-implantation embryos. The central lines are the median of data. The lower and upper hinges correspond to the 25th and 75th percentiles. The end of the lower and upper whiskers are 1.5 * IQR (inter-quartile range). Data beyond the end of the whiskers are plotted as outliers. **e,** Genome browser view of *Dlk1-Dio3* methylation profiles of WT-AG-haESCs and DKO-AG-haESCs (GSE60076[6] and GSE168447[7]). The red-shaded box indicates the *IG/Gtl2* DMRs. The yellow-shaded box indicates the promoter regions containing miRNAs. Green bars are the same as those in **a**. Resolution, 50kb. **f,** Volcano plot shows the differently expressed miRNAs of DKO-AG-haESCs compared to WT-AG-haESCs[8] in all miRNAs. *P*-values were calculated by Deseq2; *P*-value < 0.05. **g,** Bar plot shows the expression of *Dlk1-Dio3* miRNAs between WT-AG-haESCs and DKO-AG-haESCs. *P*-values were determined by Student's two-sided paired *t*-test. **h,** Volcano plots show the differently expressed miRNAs of SC embryos from WT-AG-haESCs compared to *Dnmt*-null SC embryos. *P*-values were calculated by Deseq2; *P*-value < 0.05. **i,** Bar plot shows the expression of *Dlk1-Dio3* miRNAs between SC embryos from DKO-AG-haESCs and *Dnmt*-null SC embryos. The total RNA used in **h** and **i** extracted from the E6.5 SC epiblast. *P*-values were determined by Student's two-sided paired *t*-test. **j,** Expression of *let-7b-5p* in SC embryos from DKO-AG-haESCs and *Dnmt*-null SC embryos. Data are mean ± s.e.m of three biological replicates. Cpm means counts per million. *P*-values were calculated by Deseq2. All source data are provided as a Source Data file.

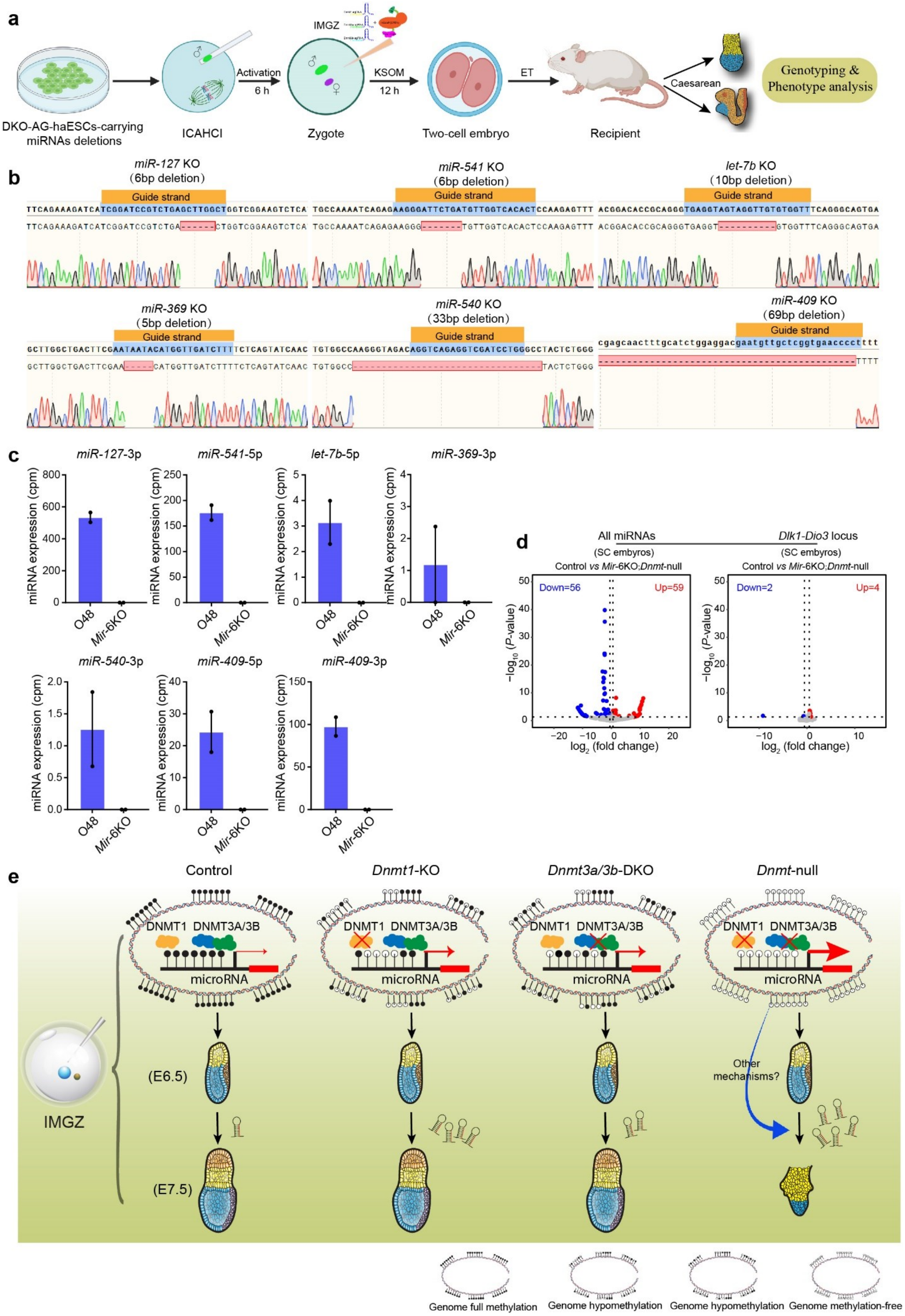
a
IMGZ
DKO-AG-haESCs-carrying miRNAs deletions
ICAHCI
Activation
6 h
Zygote
KSOM
12 h
Two-cell embryo
ET
Recipient
Caesarean
Genotyping & Phenotype analysis
b
miR-127 KO
(6bp deletion)
Guide strand
miR-541 KO
(6bp deletion)
let-7b KO
(10bp deletion)
miR-369 KO
(5bp deletion)
miR-540 KO
(33bp deletion)
miR-409 KO
(69bp deletion)
c
miR-127-3p
miR-541-5p
let-7b-5p
miR-369-3p
miR-540-3p
miR-409-5p
miR-409-3p
miRNA expression (cpm)
O48
Mir-6KO
d
All miRNAs
Dlk1-Dio3 locus
(SC embryos)
Control vs Mir-6KO;Dnmt-null
Down=56
Up=59
Down=2
Up=4
−log10 (P-value)
log2 (fold change)
e
Control
Dnmt1-KO
Dnmt3a/3b-DKO
Dnmt-null
DNMT1
DNMT3A/3B
microRNA
(E6.5)
(E7.5)
IMGZ
Other mechanisms?
Genome full methylation
Genome hypomethylation
Genome hypomethylation
Genome methylation-free

**Supplementary Fig. 19 | Dosage of *Dlk1-Dio3* miRNAs may play roles in gastrulation.**

**a,** Schematic diagram of strategies to investigate the function of miRNA dosage in gastrulation based on AG-haESC-mediated semi-cloning (SC) technology (created with BioRender.com). **b,** Chromatograms of Sanger sequencing confirm miRNA deletions in a DKO-AG-haESCs (also termed O48[8]), including *miR-127*, *miR-541*, *let-7b*, *miR-369*, *miR-540*, and *miR-409*. **c,** Expression of *miR-127-3p*, *miR-541-5p*, *let-7b-5p*, *miR-369-3p*, *miR-540-3p*, *miR-409-5p*, and *miR-409-3p* in O48 and *Mir*-6KO O48. Cpm means counts per million. Two independent repeats for each sample. Bar plots show the mean from two experimental replicates. **d,** Volcano plots show the differently expressed *Dlk1-Dio3* miRNAs between SC embryos from O48 compared and *Mir*-6KO SC embryos carrying *Dnmt*-null through the IMGZ system. The total RNA was extracted from the epiblast of SC embryos at E7.5. *P*-values were calculated by Deseq2; $Log_2$ (fold change) > 0.5; *P*-value < 0.05. **e,** Summary of DNA methylation-mediated inhibition of miRNA expression in gastrulation revealed by IMGZ. The genome DNA methylation is completely lost in *Dnmt*-null embryos, stirring widespread effects on the transcriptome and epigenome. Some unknown mechanisms combined with upregulated miRNAs in the methylation-free embryos control embryo gastrulation. All source data are provided as a Source Data file.

**Supplementary References**


1 Argelaguet, R. et al. Multi-omics profiling of mouse gastrulation at single-cell resolution. Nature 576, 487-491, doi:10.1038/s41586-019-1825-8 (2019).

2 Zhang, Y. et al. Dynamic epigenomic landscapes during early lineage specification in mouse embryos. Nat Genet 50, 96-105, doi:10.1038/s41588-017-0003-x (2018).

3 Treiber, T. et al. A Compendium of RNA-Binding Proteins that Regulate MicroRNA Biogenesis. Molecular cell 66, 270-284.e213, doi:10.1016/j.molcel.2017.03.014 (2017).

4 Wang, L. et al. Programming and inheritance of parental DNA methylomes in mammals. Cell 157, 979-991, doi:10.1016/j.cell.2014.04.017 (2014).

5 Yang, Q. et al. Highly sensitive sequencing reveals dynamic modifications and activities of small RNAs in mouse oocytes and early embryos. Science advances 2, e1501482, doi:10.1126/sciadv.1501482 (2016).

6 Zhong, C. et al. CRISPR-Cas9-Mediated Genetic Screening in Mice with Haploid Embryonic Stem Cells Carrying a Guide RNA Library. Cell Stem Cell 17, 221-232, doi:10.1016/j.stem.2015.06.005 (2015).

7 Zhang, H. et al. Epigenetic integrity of paternal imprints enhances the developmental potential of androgenetic haploid embryonic stem cells. Protein & cell 13, 102-119, doi:10.1007/s13238-021-00890-3 (2022).

8 Li, Q. et al. Temporal regulation of prenatal embryonic development by paternal imprinted loci. Sci China Life Sci 63, 1-17, doi:10.1007/s11427-019-9817-6 (2020).
