# Supplementary material for: Base editing-mediated one-step inactivation of the Dnmt gene family reveals critical roles of DNA methylation during mouse gastrulation
Source: Nat Commun. 2023 May 22;14:2922. doi: 10.1038/s41467-023-38528-z (PMC10203112; doi:10.1038/s41467-023-38528-z)
Supplement: Supplementary file 3 — Description of Additional Supplementary Files [file 41467_2023_38528_MOESM3_ESM.pdf]

Description of Additional Supplementary Files

Title: Supplementary Data 1
Description: Results of Blunt cloning and sequencing analysis of targeted *EGFP* and *Crygc* in the blastocysts.

Title: Supplementary Data 2
Description: Summary of SNVs, indels, and non-synonymous mutations identified by WGS.

Title: Supplementary Data 3
Description: The output of LC-MS quantification of 5mC and 5hmC nucleosides in *Tet* and *Dnmt* mutant embryos.

Title: Supplementary Data 4
Description: Blunt cloning and sequencing results of targeted *Tet* and *Dnmt* genes in the blastocysts derived after hA3A-eBE3-Y130F-mediated editing.

Title: Supplementary Data 5
Description: Deep sequencing analysis of *Tet*-TKO, *Dnmt1*-KO, *Dnmt3a/3b*-DKO, and *Dnmt*-null embryos.

Title: Supplementary Data 6
Description: Efficiencies of embryonic development and genome editing after IMGZ-treatment.

Title: Supplementary Data 7
Description: Deep sequencing analysis of *Tet* and *Dnmt* mutant embryos.

Title: Supplementary Data 8
Description: The quality control of WGBS data in this study.

Title: Supplementary Data 9
Description: Lists of primers and sgRNA sequences in this study.
