# Supplementary material for: Base editing-mediated one-step inactivation of the Dnmt gene family reveals critical roles of DNA methylation during mouse gastrulation
Source: Nat Commun. 2023 May 22;14:2922. doi: 10.1038/s41467-023-38528-z (PMC10203112; doi:10.1038/s41467-023-38528-z)
Supplement: Supplementary file 13 — Reporting Summary [file 41467_2023_38528_MOESM13_ESM.pdf]

nature portfolio

Corresponding author(s): Jinsong Li

Last updated by author(s): April 17, 2023

Reporting Summary

Nature Portfolio wishes to improve the reproducibility of the work that we publish. This form provides structure for consistency and transparency in reporting. For further information on Nature Portfolio policies, see our Editorial Policies and the Editorial Policy Checklist.

Statistics

For all statistical analyses, confirm that the following items are present in the figure legend, table legend, main text, or Methods section.

n/a	Confirmed	
☐	☒	The exact sample size (*n*) for each experimental group/condition, given as a discrete number and unit of measurement
☐	☒	A statement on whether measurements were taken from distinct samples or whether the same sample was measured repeatedly
☐	☒	The statistical test(s) used AND whether they are one- or two-sided<br>*Only common tests should be described solely by name; describe more complex techniques in the Methods section.*
☐	☒	A description of all covariates tested
☒	☐	A description of any assumptions or corrections, such as tests of normality and adjustment for multiple comparisons
☐	☒	A full description of the statistical parameters including central tendency (e.g. means) or other basic estimates (e.g. regression coefficient) AND variation (e.g. standard deviation) or associated estimates of uncertainty (e.g. confidence intervals)
☐	☒	For null hypothesis testing, the test statistic (e.g. *F*, *t*, *r*) with confidence intervals, effect sizes, degrees of freedom and *P* value noted<br>*Give P values as exact values whenever suitable.*
☒	☐	For Bayesian analysis, information on the choice of priors and Markov chain Monte Carlo settings
☒	☐	For hierarchical and complex designs, identification of the appropriate level for tests and full reporting of outcomes
☐	☒	Estimates of effect sizes (e.g. Cohen's *d*, Pearson's *r*), indicating how they were calculated

Our web collection on statistics for biologists contains articles on many of the points above.

Software and code

Policy information about availability of computer code

Data collection	Images were collected using OLYMPUS SZX16 microscope.<br>Western blot signals were detected using Mini Chemiluminescent Imaging and Analysis System.<br>BD FACS Aria II collected the FACS data.<br>The LC–MS/MS analysis were performed using a UPLC system (1290 series, Agilent Technologies) coupled to a triple quadrupole mass spectrometer (Agilent 6495 QQQ, AgilentTechnologies).
Data analysis	SnapGene (Version 2.3.3) was used to analyze the Sanger sequencing results.<br>GraphPad Prism 7 and Microsoft Office Excel (office 2016) were used to perform statistical analyses.<br>FlowJo_V10 was used to process FACS data.<br>Image J was used to handle the images.<br>Original bisulfite sequencing data was analyzed by the DNA methylation analysis tool (http://services.ibc.uni-stuttgart.de/BDPC/BISMA/)<br>Data processing of WGBS: Trim_galore (v0.6.4) (https://github.com/FelixKrueger/TrimGalore) was used to remove low-quality reads and adaptor sequences. Reads pass quality control were mapped to mm10 mouse genome using BitMapperBS (v1.0.2.2) in pair-end mode with parameters "--sensitive --pbat --unmapped_out" at first. Unmapped reads were output by samtools (v1.11) and mapped again by using BitMapperBS's single-end mode. Aligned bam files were merged and sorted by samtools and the methylation information in MethylKit and bedGraph format were extracted by MethylDackel (v0.4.0) (https://github.com/dpryan79/MethylDackel) with default parameters. Bed format methylation files were generated by local scripts.<br>Data processing of RNA-seq: Trim galore (v0.6.4) was used to quality control for raw fastq data. After that, clean reads were mapped to the mm10 genome by STAR (2.7.2b) with parameters "-quant Mode Transcriptome SAM Gene Counts" to output alignments translated into transcript coordinates in the bam files and read counts of each gene, which were then normalized to TPM (Transcript Per Millions) values. To ensure data quality, only replicates with Pearson's correlation greater than 0.9 were retained for further analysis. Gene-level count matrices

were imported and performed differential expression analysis by R package DESeq2 (v1.26.0).
Retrotransposons expression analysis: In detailed, unique and multiple mapped reads were assigned to class and family levels UCSC RepeatMasker annotation using featureCounts (v2.0.0) package with parameters '-M --fraction', which weight multi-mapping reads by total alignments. Differentially expressed retrotransposons were identified by DESeq2 (v1.26.0) with fold change > 2 and adjusted P-value < 0.05.
miRNA data processing: The adapter sequences were removed from the 3' end of the sequenced reads using the fastp (version 0.20.1) with parameters "-l 15 --length_limit 40". The remaining reads were mapped to the mouse genome (version GRCm38) using bowtie (version 1.2.1.1) first. The mapped reads were mapped again to the mature miRNA sequences annotated in miRbase (version 21), allowing -2 or +2 nt to be templated by the corresponding genomic sequence at the 3' end to determine the expression level of each miRNA. The differential expression analysis of each miRNA was performed by R package DESeq2 (version 1.30.1).

For manuscripts utilizing custom algorithms or software that are central to the research but not yet described in published literature, software must be made available to editors and reviewers. We strongly encourage code deposition in a community repository (e.g. GitHub). See the Nature Portfolio guidelines for submitting code & software for further information.

Data

Policy information about availability of data

All manuscripts must include a data availability statement. This statement should provide the following information, where applicable:
- Accession codes, unique identifiers, or web links for publicly available datasets
- A description of any restrictions on data availability
- For clinical datasets or third party data, please ensure that the statement adheres to our policy

The whole genome sequencing data of E11.5 embryos data generated in this study have been deposited in the NCBI Sequence Read Archive database under accession code (PRJNA574624). The deep sequencing data are deposited in the Gene Expression Omnibus (GEO) under the accession code GSE188658. The genome-wide methylation sequencing and RNA sequencing data from this study have been deposited in the GSE162903. The small RNA sequencing data have been deposited in the GSE205563. The original data presented in graphs generated in this study are provided in the Source Data file. Some source data are provided with this paper and have been deposited in the figshare database under accession code 22580038.

Research involving human participants, their data, or biological material

Policy information about studies with human participants or human data. See also policy information about sex, gender (identity/presentation), and sexual orientation and race, ethnicity and racism.

Reporting on sex and gender	N/A.
Reporting on race, ethnicity, or other socially relevant groupings	N/A.
Population characteristics	N/A.
Recruitment	N/A.
Ethics oversight	N/A.

Note that full information on the approval of the study protocol must also be provided in the manuscript.

Field-specific reporting

Please select the one below that is the best fit for your research. If you are not sure, read the appropriate sections before making your selection.

☒ Life sciences ☐ Behavioural & social sciences ☐ Ecological, evolutionary & environmental sciences

For a reference copy of the document with all sections, see nature.com/documents/nr-reporting-summary-flat.pdf

Life sciences study design

All studies must disclose on these points even when the disclosure is negative.

Sample size	No statistical method was used to predetermine sample size. For the embryo experiments, the sample size showed in the figure legends or data source file. For most experiment, we used n>=3 biological replicates size to confirm our results. Results obtained were highly significant and consistent and did not require larger animal experimental groups.
Data exclusions	No data were excluded from the analysis.
Replication	All replication attempts were successful. The size of biological replication was shown in the legends.
Randomization	Randomization was not applicable to this study. In all animal studies, mice were grouped by genotype.

Blinding	The same investigator set up and analyzed the experiments, no blinding was performed. Unbiased analysis of data was carried out wherever possible.

Reporting for specific materials, systems and methods

We require information from authors about some types of materials, experimental systems and methods used in many studies. Here, indicate whether each material, system or method listed is relevant to your study. If you are not sure if a list item applies to your research, read the appropriate section before selecting a response.

Materials & experimental systems

n/a	Involved in the study
☐	☒ Antibodies
☐	☒ Eukaryotic cell lines
☒	☐ Palaeontology and archaeology
☐	☒ Animals and other organisms
☒	☐ Clinical data
☒	☐ Dual use research of concern
☒	☐ Plants

Methods

n/a	Involved in the study
☒	☐ ChIP-seq
☐	☒ Flow cytometry
☒	☐ MRI-based neuroimaging

Antibodies

Antibodies used	The following antibodies were used for western blot: anti-DNMT1 (1:2000 dilution, Cell Signaling Technology, Cat#5032S), anti-DNMT3A (1:2000 dilution, Proteintech, Cat#20954-1-AP), anti-DNMT3B (1:2000 dilution, Abcam, Cat#ab2851),and anti-GAPDH (1:5000 dilution, Proteintech, Cat# 60004-1-Ig), HRP-conjugated anti-rabbit (1:5000 dilution, BBI, Cat#D110058-0001), and anti-mouse antibody (1:10,000 dilution, Proteintech, Cat#SA00001-1).
Validation	anti-DNMT1 (1:2000 dilution, Cell Signaling Technology, Cat#5032S): (Specificity were confirmed in this study and other studies (PMID: 36414620; PMID: 36351409; PMID: 35906225; PMID: 35906225), by manufacturer (https://www.cellsignal.com/products/primary-antibodies/dnmt1-d63a6-xp-rabbit-mab/5032?site-search-type=Products&N=4294956287&Ntt=5032s&fromPage=plp&_requestid=883879).<br>anti-DNMT3A (1:2000 dilution, Proteintech, Cat#20954-1-AP): (Specificity were confirmed in this study and other studies (PMID: 35080973; PMID: 34865203; PMID: 32522567; PMID: 36536414), by manufacturer (https://www.ptglab.com/products/DNMT3A-Antibody-20954-1-AP.htm).<br>anti-DNMT3B (1:2000 dilution, Abcam, Cat#ab2851): (Specificity were confirmed in this study and other studies (PMID: 29456180; PMID: 30617255; PMID: 32483152; PMID: 32265226), by manufacturer (https://www.abcam.com/products/primary-antibodies/dnmt3b-antibody-ab2851.html).<br>anti-GAPDH (1:5000 dilution, Proteintech, Cat# 60004-1-Ig): (Specificity were confirmed in this study and other studies (PMID: 36477534; PMID: 33177715; PMID: 34040255; PMID: 35511979), by manufacturer (https://www.ptglab.com/products/GAPDH-Antibody-60004-1-Ig.htm).

Eukaryotic cell lines

Policy information about cell lines and Sex and Gender in Research

Cell line source(s)	One WT-AG-haESC and two DKO-AG-haESC lines (408 and O48) were established in our previous study (Yang, H. et al. Cell.149 (2012): 605-617; Zhong, C. et al. Cell Stem Cell.17(2015):221-232). One DKO-AG-haESC was named 408 in the paper (Li, Q. et al. Nature Cell Biology.20 (2018):1315-1325). One DKO-AG-haESC was named O48 in the paper (Li, Q. et al. Sci China Life Sci. 63 (2020):1-17).
Authentication	One WT-AG-haESC and two DKO-AG-haESC lines (408 and O48) were established by our lab and have no authentication.
Mycoplasma contamination	Cell lines were tested to be free of mycoplasma contamination.
Commonly misidentified lines (See ICLAC register)	No commonly misidentified cell lines used in this study.

Animals and other research organisms

Policy information about studies involving animals; ARRIVE guidelines recommended for reporting animal research, and Sex and Gender in Research

Laboratory animals	Mice were housed in individually ventilated cages (IVC) under a credited specific pathogen-free facility. The room has controlled temperature (20-25 °C), humidity (30-70 %) and light (12 h light-dark cycle). Zygotes are all collected from eight weeks female B6D2F1 (C57BL/6♀× DBA2♂) mice, which were mated to ten-twenty weeks Oct4-EGFP males (C57BL/6 background). The ten weeks mT/mG males (full name B6.129(Cg)-Gt(ROSA)26Sor tm4(ACTB-tdTomato,-EGFP)Luo/J; JAX strain 007676) were used to evaluate the off-target effect of hA3A-eBE-Y130F. The ten-fourteen weeks ICR females were used as pseudo-pregnant foster mothers. The strains

	of Stra8-Cre and Zp3-Cre mice at 8-15 weeks used in this study were FVB/N-Tg (Stra8-Cre)1Reb32 and C57BL/6-TgN (Zp3-Cre) 93Knw33, respectively. Dnmt12lox, Dnmt3a2lox and Dnmt3b2lox mouse strains at 10-14 weeks were described previously (Jackson-Grusby, L. et al. Nature Genetics. (27):31-39; Kaneda, M. et al. Nature.(429):900-903).
Wild animals	The study did not involve wild animals.
Reporting on sex	Zygotes are all collected from eight weeks female B6D2F1 mice, which were mated to ten-twenty weeks Oct4-EGFP males.
Field-collected samples	The study did not involve field-collected samples.
Ethics oversight	All animal procedures were approved by the Institutional Animal Care and Use Committee of the Center for Excellence in Molecular Cell Science, Chinese Academy of Sciences.

Note that full information on the approval of the study protocol must also be provided in the manuscript.

Flow Cytometry

Plots

Confirm that:

☒ The axis labels state the marker and fluorochrome used (e.g. CD4-FITC).

☒ The axis scales are clearly visible. Include numbers along axes only for bottom left plot of group (a 'group' is an analysis of identical markers).

☒ All plots are contour plots with outliers or pseudocolor plots.

☒ A numerical value for number of cells or percentage (with statistics) is provided.

Methodology

Sample preparation	For GOIT, E11.5 embryos were dissociated by 1 ml 0.25% Trypsin-EDTA (Gibco, Cat#25200056) in 37 °C water bath for 20-30 min, constantly blowing with a 1ml pipette tips. The digestion was terminated by adding 1ml DMEM medium with 10% FBS (Excell Bio). The cell suspension was centrifuged at 1200 rpm for 6 min, rinsed one time with DPBS, transferred through a 40-µm cell strainer.<br>For AG-haESCs, haploid stem cell clones were dissociated into single cells with 0.05% Trypsin-EDTA in 37 °C for 5min, and then incubated with 15 µg/ml Hoechst 33342 in 37 °C water bath for 8 min. Then, cell suspension was filtered with 40-µm cell strainer before sorting.
Instrument	BD FACS Aria II
Software	FlowJo_V10 was used to analyze FACS data.
Cell population abundance	The single cell suspension from E11.5 embryos was sorted in BD FACS Aria II with purity of priority. The tdtomato+and EGFP+ cells were enriched, separately.<br>The single cell suspension of AG-haESCs was sorted in BD FACS Aria II with purity of priority. The double positive cells (mCherry positive and the first peak of DAPI) were enriched only.
Gating strategy	For GOIT, EGFP and RFP positive boundaries were determined by positive and negative samples, which were derived from Cre-injected and un-injected groups, respectively.

☒ Tick this box to confirm that a figure exemplifying the gating strategy is provided in the Supplementary Information.
